# Spinning Green: Lipase-Catalyzed Synthesis of Bioactive Fatty Acid Amides from Renewable Lipid Feedstocks in a Rotating Bed Reactor

**DOI:** 10.1021/acssuschemeng.5c07654

**Published:** 2025-10-13

**Authors:** Martina Bigliardi, Silvia Donzella, Diana-Ionela Dăescu, Alessandro Pellis, Lucia Tamborini, Andrea Pinto, Martina L. Contente

**Affiliations:** † 9304 University of Milan, Department of Food, Nutrition and Environmental Sciences (DeFENS), Via Celoria 2, Milan 20133, Italy; ‡ Politehnica University Timişoara, Faculty of Chemical Engineering, Biotechnologies and Environmental Protection, Vasile Pârvan 6, Timişoara 300223, Romania; § 9302 University of Genoa, Department of Chemistry and Industrial Chemistry, Via Dodecaneso 31, Genova 16146, Italy; ∥ 9304 University of Milan, Department of Pharmaceutical Sciences (DISFARM), Via Mangiagalli 25, Milan 20133, Italy

**Keywords:** fatty amides, biocatalysis, fermentation technology, spinchem reactor, lipase, (bio)process intensification, green solvents

## Abstract

Fatty acid ethanolamides (FAEAs) are bioactive lipids involved in inflammation, pain modulation, and energy homeostasis, gaining interest in the pharmaceutical, nutraceutical, and cosmetic sectors. Here, we present an intensified biocatalytic strategy for the synthesis of a mixture of FAEAs—palmitoylethanolamide (PEA), oleoylethanolamide (OLA), stearoylethanolamide (SEA), and linoleoylethanolamide (LEA)—starting from microbial lipids extracted from*Cutaneotrichosporon oleaginosus*, cultivated on whey permeate, a major dairy byproduct, supplemented with waste cooking oil. The two-step enzymatic cascade—transesterification of triacylglycerols into ethyl esters followed by aminolysis with ethanolamine—was catalyzed by Novozym 435 (immobilized *Candida antarctica* lipase B) in green solvents. Whereas ethanol has been used for the first step, eucalyptol proved particularly effective in aminolysis reaction with >99% conversion and complete selectivity. Process intensification *via* a SpinChem rotating bed reactor led to a 5-fold reduction in reaction time (48 to 10 h), a 5- to 7-times increase in space-time yield and quantitative yields for both steps. Biocatalyst reusability and process reproducibility was preserved. This integrated platform exemplifies a circular bioeconomy approach by valorizing agri-industrial residues into high-value compounds through clean and scalable technologies. The resulting FAEAs hold potential for synergistic therapeutic applications, while supporting cost-effective and sustainable manufacturing across diverse industries.

## Graphical abstract

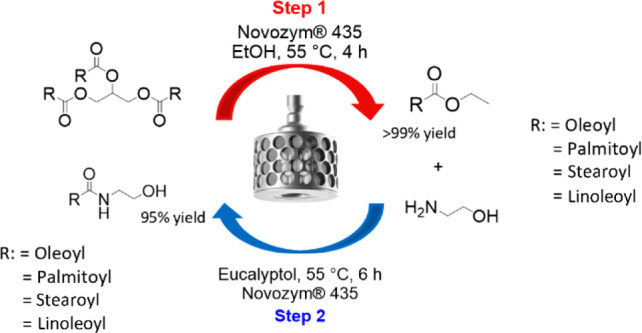

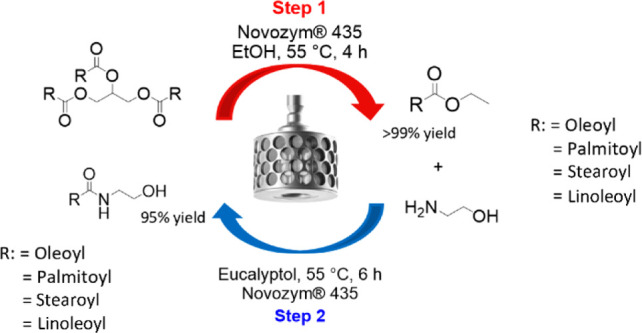

## Introduction

Fatty amides are naturally occurring signaling molecules found in various plant and animal tissues, where they play a key role in physiological processes.^1^

Among fatty amides, fatty acid ethanolamides (FAEAs) (Figure 1) represent a crucial class of endogenous lipid mediators involved in the regulation of inflammation, energy homeostasis, appetite and pain perception.^2^ Palmitoylethanolamide (*N*-palmitoylethanolamine or PEA)—the progenitor of this family—was first identified as an immunomodulatory molecule in 1957 by Nobel laureate Rita Levi-Montalcini.^3^ Since then, PEA has shown promising multitarget therapeutic potential, particularly for inflammation, neurodegenerative disorders, and neuropathic pain.^3^

**Figure 1:**
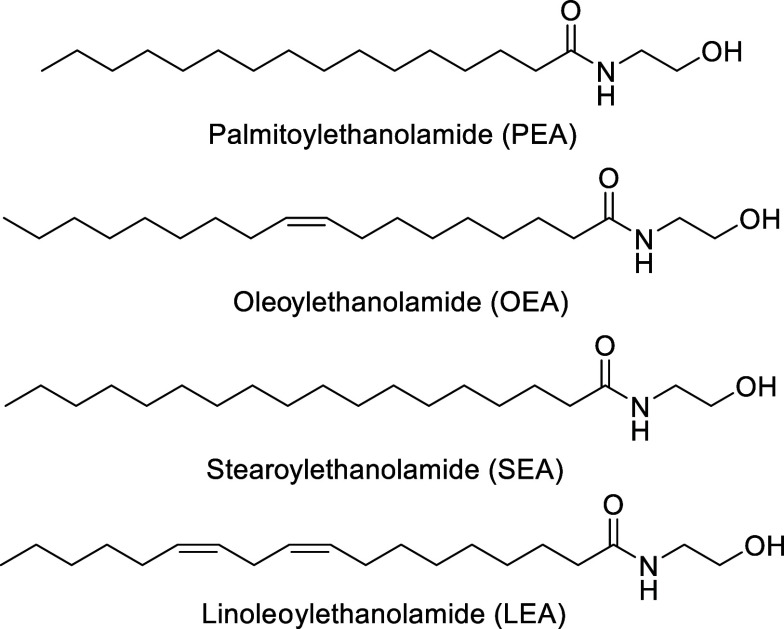
Structure of representative biologically active fatty acid ethanolamides (FAEAs).

Modern formulations of PEA are often coadministered with antioxidant compounds such as luteolin, polydatin, quercetin, as well as other FAEAs like oleoylethanolamide (*N*-oleylethanolamine, OEA, Figure 1), stearoylethanolamide (*N*-stearoylethanolamine, SEA, Figure 1), as well as linoleoylethanolamide (*N*-linoleoylethanolamine, LEA, Figure 1), enhancing their pharmacological profile.^4^ Notably, while (OEA)-enriched preparations have received FDA (*i.e.*, Food and Drug Administration) approval as dietary supplements for appetite regulation, weight management, and cholesterol control, SEA has demonstrated anti-inflammatory, pro-apoptotic and anorexigenic effects, supporting its potential application in the treatment of neuroinflammation-related disorders.^5,6^ Similarly, LEA has been reported to exhibit several bioactivities comparable to those of its analogues, including involvement in the regulation of food intake and neuroprotection.^7,8^

To meet the growing demand for bioactive FAEAs, both chemical and biocatalytic synthetic strategies have been explored. Conventional synthetic procedures commonly rely on toxic coupling agents—such as 1-ethyl-3-(3-(dimethylamino)­propyl)­carbodiimide (EDC) or *N,N’*-dicyclohexylcarbodiimide (DCC)—to activate carboxylic acids before reacting with ethanolamine. However, these approaches generate substantial waste, raise safety concerns, and often require protection/deprotection steps that reduce atom economy.^9^ Other methods, such as direct amidation of esters using Mg­(OCH_3_)_2_ and CaCl_2_ or metal catalysts, frequently suffer from low conversion, poor selectivity, long reaction times, and challenging purifications.^10^ Moreover, the harsh conditions typically employed—high temperatures, inert atmospheres, hazardous solvents—can negatively affect the physicochemical properties of the final products thus reducing their biological activity.^11^

In contrast, biocatalysis offers a powerful and sustainable alternative characterized by exceptional chemo- and regio-selectivity under mild, environmentally benign conditions.^12^ Among the available biocatalysts, lipases (EC 3.1.1.3) have proven particularly effective in catalyzing esterification, transesterification, and aminolysis reactions across a wide range of substrates. Their compatibility with natural starting materials and operation in both aqueous and organic media make them particularly suited for green synthesis.^13–16^ Beyond single-step reactions, lipases, are increasingly applied in multistep and cascade processes, where sequential or concurrent transformations are integrated within a single framework. Such strategies reduce the need for intermediate purification, improve atom economy, and streamline synthetic routes to valuable products.^17–20^

To further enhance the performance of enzyme catalysis, innovative strategies such as process engineering can be employed.^21^ Advanced process-intensification technologies, like rotating bed reactors (RBRs), offer significant benefits by addressing solubility issues, boosting biocatalyst efficiency, and streamlining manufacturing workflows.^21–23^ Among them, the SpinChem RBR is designed to optimize mass and heat transfer significantly reducing reaction times and improving the overall productivity. Its cylindrical basket, protecting the immobilized biocatalyst from mechanical stress, prolongs its operational lifespan and facilitate its recovery and reuse.^24^ These advantages, together with simplified downstream steps (*e.g.,* no need for filtration) make SpinChem RBR highly attractive for scalable, sustainable and cost-effective manufacturing.^24^

Aligned with the European Green Deal and the Sustainable Development Goals, there is increasing emphasis on clean technologies and circular economy models, including the valorization of agri-food waste.^25^ In this framework, biocatalysis represents a key technology to convert low-value residues into high-value chemicals, biomaterials and bioactive compounds, contributing to both environmental sustainability and industrial innovation.^26–28^

Among food industry byproducts, cheese whey (CW) is one of the most abundant in Europe. In Italy alone, approximately 6 million tons are generated annually, with nearly 10 L of CW produced every kilogram of cheese.^29^ Rich in lactose (45–50 g/L), proteins (6–8 g/L), lipids (4–5 g/L), and essential minerals such as calcium, potassium, and phosphorus, CW represents a valuable nutrient-rich resource with significant potential for valorization. Through membrane filtration, proteins can be efficiently recovered, yielding whey permeate (WP), which retains fermentable sugars and nutrients suitable for microbial fermentation.^30^ Our group established expertise in cultivating oleaginous yeasts on food waste-derived substrates as sustainable feedstocks.^27,30^ In addition, waste cooking oil (WCO), which exceeded 200 million tons per year in 2025, is an established source of carbon and nitrogen used to induce or boost lipase production but also suitable to support the growth of industrial microorganisms.^31,32^

In particular, *C. oleaginosus* ATCC 20509 has demonstrated remarkable lipid accumulation capabilities storing intracellular triacylglycerols (TAGs) mainly employed in biodiesel and oleochemical production.^27^ These lipids primarily consist of long-chain fatty acids—oleic (C18:1), palmitic (C16:0), stearic (C18:0), and linoleic acid (C18:2)—which serve as ideal precursors for the synthesis of bioactive FAEAs.^30^

In the context of green chemistry, replacing traditional petroleum-based solvents with environmentally friendly alternatives is a key aspect for sustainable biotransformations. While water remains the solvent of choice for many enzymatic reactions, the use of organic solvents is often necessary to address solubility limitations, increase substrate loading, or shift reaction equilibria toward condensation over hydrolysis. In such cases, solvent-free systems and the adoption of biobased green solvents offer safer and more sustainable solutions, minimizing environmental and health impacts.^33^ Significant research efforts have been devoted to identifying and developing greener alternatives resulting in a growing portfolio of bioderived solvents with physical properties comparable to conventional organic solvents.^33–35^

In this work, we present an intensified biocatalytic process for the selective synthesis of bioactive fatty acid amides PEA, OLA, SEA and LEA from ethanolamine and lipid-rich extracts derived from waste-grown *C. oleaginosus*. Ethanolamine was selected as the nucleophile both for the relevance of the resulting products and to demonstrate the complete regioselectivity of the catalyst. The process employs Novozym 435—an immobilized form of *Candida antarctica* lipase B—operated in a SpinChem rotating bed reactor to enhance reaction efficiency and catalyst reusability. To improve the environmental profile of the system, a range of renewable and low-toxicity solvents—including phenetole (PHE), anisole (ANI), 2-methyltetrahydrofuran (2-MeTHF), 2,2,5,5-tetramethyloxolane (TMO), and eucalyptol (EUC)—were evaluated for their compatibility with the lipase-catalyzed amidation reaction. Among them, EUC emerged as the most effective option, enabling high conversion and excellent selectivity under mild reaction conditions. Derived from natural sources, EUC combines favorable physicochemical properties with low toxicity and biodegradability, making it an ideal solvent for the development of greener biocatalytic processes.^33^ This integrated strategy ultimately enabled the efficient production of a defined mixture of high-value bioactive FAEAs—PEA, OLA, SEA and LEA—paving the way for synergistic biobased formulations with enhanced therapeutic potential.

## Experimental Section

### General Information

Chemicals and solvents were obtained from Merck Life Science srl (Milan, Italy) and used without further purification. Specifically, ethanol, PHE, ANI, 2-MeTHF, and EUC were purchased with a purity >99%. TMO was synthesized according to a previously reported procedure,^33,36^ achieving a purity >98% (see Supporting Information). TMO was characterized by ^1^H NMR (JEOL ECZ400R/S3, 400 MHz; chemical shifts (*δ)* in ppm, coupling constants (*J)* in Hz) and GC–MS (Shimadzu GC-2010 Plus equipped with an AOC-20i Plus autoinjector and an Avantor Hichrom HI-5 MS column, 30 m × 0.25 mm internal diameter, 0.25 μm film thickness). The GC was coupled to a Shimadzu GCMS-QP2010SE mass spectrometer. Analyses were performed by injecting 1 μL in split mode (1:10 split ratio), with the injector temperature set at 250 °C and helium as carrier gas (1 mL/min). The oven temperature program was: hold at 50 °C for 4 min, increase to 300 °C at 10 °C/min, and hold for 4 min at 300 °C. Whenever possible, solvents were purchased in dried form; otherwise, they were stored for 24 h over preactivated molecular sieves (oven-dried at 115 °C overnight). Cell growing and strain maintaining media were bought from Thermo Fischer Scientific (Segrate, Italy) or Biolife (Milan, Italy). Whey permeate was provided Latteria Soresina (Soresina, Cremona, Italy), WCO was a mixture of vegetable fried oil (soybean, peanut and sunflower oils), while candied mango syrup for feeding operation was received from SVZ (Industrial Fruit and Vegetable Ingredients, Breda, The Netherlands). Merck Silica gel 60 F254 (aluminum foil) plates were used for TLC analysis; flash column chromatography was performed on Merck Silica gel (230–400 mesh) Merck Life Science srl (Milan, Italy). Detection of TLC analyses have been performed using the cerium molybdate as a stain solution, prepared as follows: 12 g of ammonium molybdate, 0.5 g of ceric ammonium molybdate, 15 mL of concentrated sulfuric acid and 235 mL of water, and developed by heating. *Candida antarctica* lipase B (CaLB) immobilized onto acrylic resin (Novozym 435) was purchased from Merck Life Science srl (Milan, Italy) with a supplier declared activity of ≥ 5000 PLU g^–1^. For yeast fermentation a 2-L bioreactor has been employed (ez2-Control from Applikon Biotechnology, Delft, The Netherlands) equipped with an AppliSens oxygen probe and an AppliSens pH electrode for continuous parameter monitoring. The spectrophotometric assays were carried out by employing Eppendorf BioPhotometerD30 Spectrophotometer. The scaled-up reactions were performed in a SpinChem RBR S2 reactor (Umeå, Sweden) which was connected to a WB thermostatic bath (Steroglass Srl, Perugia, Italy) equipped with an ARGOLab WB 22 recirculation pump for perfect temperature uniformity. NMR spectra of FAEEs and FAEAs were recorded on Bruker AvanceTM NEO 400 MHz employing the residual signal of the deuterated solvent as internal standard. Chemical shifts (δ) are expressed in ppm and coupling constants (*J*) in Hertz (Hz). GC-FID analysis we carried out using a GC-FID Shimadzu Nexis GC-2023 system equipped with a MEGA-WAX column (30 m length, 0.25 mm internal diameter and 0.25 μm film thickness). The analytical conditions were as follows: injector and detector temperature set at 250 °C; initial oven temperature 40 °C, ramped at 10 °C min^–1^ to a final temperature of 220 °C, carrier gas flow rate through the column at 1.03 mL min^– 1^; split ratio 1:10; sample concentration 1000 ppm; injection volume 1 μL. GC-MS analysis of FAEAs was performed employing a Shimadzu GC-MS QP2010 SE system equipped with a HI-5 ms column (30 m length, 0.25 mm internal diameter and 0.25 μm film thickness). GC-MS conditions: a solvent delay of 2.5 min was applied, with a mass scan range of 35–800 *m*/*z*. The injector, detector, and MS source were maintained at 250 °C. The oven temperature was initially set at 70 °C (held for 3 min), then ramped at 25 °C/min to 300 °C. The carrier gas flow rate was 1.03 mL/min with a split ratio of 1:10. Samples were analyzed at a concentration of 1000 ppm, and 1 μL was injected. LC-MS analyses were performed on a Microsaic 4000MiD mass spectrometer connected with Agilent 1100 HPLC equipped with an ACE Excel column (150 mm × 3 mm x 3 μm). LC-MS conditions: the mobile phase consisted of ultrapure water and acetonitrile with 0.1% formic acid. The gradient program was: 30% water (0 min) to 0% (15 min). The flow rate was 0.38 mL/min, with a column temperature of 30 °C. Mass spectra were acquired in positive ion mode with full scan from 100–800 *m*/*z* and a TIC voltage of 750 V. Samples were analyzed at concentrations ranging from 300–800 μg/mL, with an injection volume of 5 μL.

### Yeast Cultivation for Lipid Production

The yeast strain used in this work is*Cutaneotrichosporon oleaginosus*ATCC 20509. For long-term storage, it was maintained at −80 °C on 15% (v/v) glycerol. The media used were YPD medium containing 10 g/L yeast extract, 20 g/L peptone, 20 g/L glucose or whey permeate-based medium, composed of liquid whey permeate (stored at −20 °C until use) and supplemented with 10 g/L of yeast extract, 2.28 g/L of urea and 2% (v/v) WCO.

Precultures were performed by inoculating cells from the glycerol stocks and cultivated on YPD in baffled flasks with an air-to-liquid ratio of 5:1 at 28 °C in a rotary shaker at 150 rpm overnight. After this time, the cells were harvested by centrifugation (5000 rpm/2300 rcf, 10 min in Eppendorf 5415D centrifuge) and inoculated at OD_660_ 0.4 in the 2L-bioreactor containing 1 L of whey permeate-based medium. The temperature was set at 28 °C, the air inlet at 1 vvm, and foam formation was controlled by the addition of Sigma 204, a silicone antifoaming agent. Dissolved oxygen concentration was continuously monitored using an oxygen probe, starting from 100% saturation. A cascade control system ensured that oxygen levels remained stable, consistently above 30%. The pH was monitored using a pH electrode and automatically maintained at 6.0 ± 0.5 by the addition of 5 M KOH or 10% (v/v) H_2_SO_4_ as needed.

For cell feeding, a sterilized 1:2 diluted syrup—derived from mango-based candied fruit production and containing 199 g/L glucose and 296 g/L fructose—was used as the carbon source. At defined time points, up to 15 mL of culture was aseptically sampled using a Miniplus2 peristaltic pump (Gilson, Milan, Italy) to monitor cell growth (OD_660_), determine cell dry weight, measure residual sugar contents, and assess lipid accumulation. Sugar concentrations during fermentation were quantified using commercial enzymatic kits (K-GLUHK, K-LACGAR and K-SUFRG from Megazyme, Wicklow, Ireland). For dry cell weight determination, 2 mL of cell culture was centrifuged (10 min at 13200 rpm/16100 rcf in Eppendrof 5415D centrifuge) in preweighed Eppendorf tubes and dried overnight at 105 °C. Lipid content was measured *via* the sulfo-phospho-vanillin colorimetric assay (Spinreact, Girona, Spain) on washed cell pellets, equivalent to approximately 30 OD, resuspended in 0.5 mL of cold redistilled water as reported by Donzella et al.^30^ At the end of the process, the entire culture broth was harvested by centrifugation and freeze-dried for 24–48 h (Italian Vacuum Technology, Milan, Italy).

### Lipid Extraction from *C. oleaginosus* ATCC 20509

Yeast-derived triacylglycerols (TAGs, average MW ∼ 822 g mol^–1^) were recovered *via* an ultrasound-assisted extraction (Bandelin Sonorex Super RK 1050, 15 min) from 6 g of lyophilized biomass suspended 100 mL of *n*-heptane/*i*-PrOH (3:2 v/v). The resulting lipid fraction was subsequently centrifuged at 10000 rpm for 4 min to remove cell debris (Eppendorf Multipurpose Centrifuge 5804 R). Extraction yield (∼30%) was calculated based on the lipid content of the freeze-dried biomass (48.5% w/w), as determined by the colorimetric assay cited above.^37^

### Preparation of Ethyl Fatty Esters as Activated Acyl Moieties

#### Hydrolysis of TAGs

Enzymatic hydrolysis of TAGs was performed using 300 mg of Novozym 435 and 860 mg of extracted TAGs (equivalent to 1.05 mmol, assuming an average MW of 822 g mol^–1^). The reaction was carried out in 20 mL of 10% H_2_O in *tert*-butanol at 55 °C and 150 rpm for 24 hours.

The reaction progress was monitored by TLC using *n*-hexane:EtOAc (1:1) as a mobile phase and cerium molybdate stain solution for visualization. After completion, the reaction mixture was filtered through a sintered glass to recover the immobilized enzyme, and the filtrate was acidified with 1 M HCl solution and extracted with EtOAc (10 mL x 3). The organic layer was dried over anhydrous NaSO_4_, filtered, and concentrated under reduced pressure to yield 98% (270 mg) of FFAs (average MW ∼ 275 g mol^–1^) which were used in the subsequent step.

#### Esterification of FFAs

A mixture of FFAs (0.1 M, 0.5 mmol, average MW ∼ 275 g mol^–1^) was esterified in 5 mL of ethanol in the presence of Novozym 435 (150 mg) and molecular sieves. The reaction was carried out at 55 °C, with 150 rpm stirring, for 24 h. The reaction progress was monitored by TLC using *n*-hexane:EtOAc (8:2) as a mobile phase and cerium molybdate stain solution for visualization. Upon complete conversion the reaction mixture was filtered to recover the biocatalyst, and the solvent was removed under reduced pressure to yield the ethyl fatty ester mixture (>99% yield, with an estimated average MW of 303 g mol^–1^).

The ethyl fatty ester content was subsequently analyzed by GC as described above (see Experimental Section). Retention times: ethyl palmitate: 15.2 min, ethyl stearate: 16.9 min, ethyl oleate: 17.1 min, ethyl linoleate: 17.4 min (see Supporting Information).

#### Direct Transesterification of TAGs to Fatty Acid Ethyl Esters (FAEEs)

Extracted TAGs (0.1 M, 0.5 mmol) were suspended in 5 mL of EtOH containing molecular sieves (200 mg) under magnetic stirring (150 rpm). Novozym 435 (200 mg) was added to catalyze the complete transesterification to ethyl fatty esters at 55 °C in 24 h (>99% yield, 151 mg recovered). The reaction progress was monitored *via* TLC as reported above, while the final ethyl fatty ester profile was analyzed *via* GC as previously described (see Supporting Information).

### Batch Optimization of Lipase-Mediated Fatty Amide Synthesis and Green Solvent Selection

#### Synthesis of Fatty Amides *via* Direct Condensation of FFAs with Ethanolamine

A solution of FFAs (0.1 M, 0.1 mmol) in *n*-hexane or toluene or TMO (1 mL) was mixed with ethanolamine (1.2 equiv), molecular sieves (50 mg/100 mg) and Novozym 435 (50 mg/100 mg) at 150 rpm, 55–70 °C for 24 h. The reaction was monitored every hour by TLC analysis using EtOAc:*n*-hexane (85:15) as mobile phase and cerium molibdate as a stain. The purification was carried out *via* column chromatography (silica gel), with a gradient method EtOAc/*n*-hexane (8:2 to 100%), yielding between 25 and 30% of fatty amides (see Supporting Information).

#### Aminolysis of FAEEs with Ethanolamine

FAEEs (0.1 M, 0.1 mmol) were reacted with ethanolamine (1.2 equiv) in the presence of molecular sieves (50 mg) and Novozym 435 (50 mg) in different organic green solvents (TMO, 2-MeTHF, PHE, ANI and EUC; 1 mL each). The mixtures were stirred at 150 rpm, 55 °C for 24 h. The reactions were monitored by TLC and products were purified following the conditions described above (see Experimental Section). After purification, the isolated yields of the corresponding fatty amides were as follows: TMO: 90%, 2-MeTHF: 93%, PHE: 94%, ANI: 90%, EUC: 95% (average MW ∼ 318 g mol^–1^).

#### Process Intensification *via* SpinChem RBR

The transesterification of yeast extracted TAGs (0.1 M, 15 mmol) was carried out in a SpinChem RBR. TAGs were suspended in dry EtOH (150 mL) and transferred into the RBR vessel. The mixture was preheated to 55 °C using a thermostatic bath. The rotating internal cage was filled with 3 g of Novozym 435 and 3 g of molecular sieves (ratio 1:1). The reaction was performed at 180 rpm and monitored by TLC as previously described (see Experimental Section). After reaction completion (4 h), the reaction mixture was collected *via* the bottom valve, and the solvent was evaporated under reduced pressure. The resulting crude was dissolved in eucalyptol (150 mL), mixed with ethanolamine (1.2 equiv), and reintroduced to the RBR vessel. The cage, containing the same Novozym 435 and molecular sieves, was inserted and rotated at 150 rpm, at 55 °C. After 6 h, the reaction reached completion, and the product was recovered by solvent evaporation, as previously described. The resulting amide mixture was purified by column chromatography following the protocol reported above (see Experimental Section), affording >99% of molar conversion and 95% of isolated yield.

The resulting fatty acid amide composition was determined using GC-MS and LC-MS analyses (see Experimental Section).

## Results and Discussion

### Lipid Production by *C. oleaginosus* Grown on Whey Permeate (WP) Enriched with Waste Cooking Oil (WCO)

In line with the principles of sustainable bioprocessing and circular bioeconomy, a microbial lipid production strategy was employed to valorize WP as an abundant agri-industrial residue obtained after ultrafiltration of cheese whey (CW). While a few oleaginous yeast species have been shown to grow on CW, often after preliminary treatments such as hydrolysis and detoxification,^38^ WP is an even more challenging substrate, as most of the milk proteins have been removed. Only a very limited number of oleaginous yeasts have been tested on WP in shake-flask cultures, including*Lipomyces lipofer*DBVPG 6630,*Yarrowia lipolytica*B9,*Meyerozyma guilliermondii*BI281A,*Cystobasidium oligophagum*JRC1 and*Debaryomyces etchellsii*BM1, achieving lipid accumulation ranging from 0.4 to 7 g/L. Among them,*Cutaneotrichosporon oleaginosus*ATCC 20509 exhibited the highest productivity reaching 10 g/L.^30,39–42^ Following this strategy, in the present study *C. oleaginosus* ATCC 20509 was cultivated in a fed-batch system to promote lipid accumulation. WP was used as a low-cost, nutrient-rich medium and supplemented with urea as a cheap nitrogen source to have a significant biomass production (see Supporting Information).^30^ To further enhance carbon availability and reduce fermentation time, the medium was enriched with WCO (2% w/w), a lipid-rich residue widely available in the food service sector. At 18 and 36 h, a feed of mango syrup was necessary to ensure an unbalanced C/N ratio favorable to lipid biosynthesis (see Supporting Information). The presence of WCO enhanced process efficiency, allowing the same lipid titer (approximately 43 g/L) to be reached in only 64 h, compared to 72 h when using WP as the sole substrate.^30^ Under these optimized conditions, the final dry cell weight (DCW) reached 88.7 g/L, with intracellular lipid accumulation accounting for approximately 48.5% of DCW, corresponding to 43.5 g/L of TAGs, under nitrogen-limited conditions (see Supporting Information).

After recovery of TAGs *via* ultrasound assisted extraction the lipid fraction was converted into fatty acid ethyl esters (FAEEs) and analyzed *via* GC-FID (see Experimental Section). The fatty acid profile was predominantly composed of oleic acid (C18:1,50%), followed by palmitic (C16:0,32%), and stearic (C18:0,14%), with linoleic acid (C18:2) accounting for less than 5%. This composition is ideally suited for subsequent biocatalytic conversion into fatty acid ethanolamides (FAEAs) in particular PEA, SEA, OEA and LEA. These results confirm that the combined valorization of dairy and lipid-rich waste streams can enable efficient and accelerated microbial lipid production supporting scalable and eco-friendly routes to bio-based chemicals.

### Optimization of the Best Reaction Conditions for the Lipase-Catalyzed Synthesis of Fatty Acid Ethanolamides (FAEAs)

A thorough literature review identified the direct enzymatic amidation of carboxylic acids catalyzed by *Candida antarctica* lipase B (CaLB) in organic media, as the most promising strategy for synthesizing FAEAs (Figure 1) from the defined mixture of fatty acids (FFAs) obtained *via* hydrolysis of yeast-derived TAGs.^43–46^ However, when this approach was applied to our system, it resulted in a modest yield of only ∼ 30%. Notably, the variation of the reaction conditions such as reaction environment (*e.g., n*-hexane, toluene, TMO), catalyst loading (50–100 mg/mL) and temperature (55–70 °C) did not lead to any significant improvement in the final yield (see Supporting Information). To address this limitation, we investigated the activation of the FFAs as ethyl esters to facilitate a more efficient biocatalytic conversion. The enzymatic esterification performed in ethanol as described above (see Experimental Section), not only achieved complete conversion of FFAs into ethyl esters (FAEEs), but also provided the necessary intermediates for accurate GC analysis of the yeast lipid profile, enabling assessment of fermentation reproducibility.

Although the initial strategy involved a two-step procedure—hydrolysis of TAGs followed by esterification, both catalyzed by CaLB (see Experimental Section)—to reduce energy consumption and streamline the process, a one-step lipase-mediated transesterification of TAGs to FAEEs was developed. This direct transformation afforded full conversion in 24 hours, significantly accelerating the overall process. GC analysis confirmed that the FAEEs mixture retained the same fatty acid composition as the original FFAs, ensuring the integrity of the lipid profile throughout the process. While CaLB is generally reported to prefer long-chain alcohols in esterification mode, the reaction in ethanol proceeded efficiently.^15^ Importantly, the use of ethanol as both a substrate and a green reaction medium significantly improved the overall sustainability and atom economy of the process. The resulting FAEE mixture was subsequently subjected to aminolysis with ethanolamine (see Experimental Section). Ethanolamine was chosen as the nucleophile not only for the biological relevance of the resulting FAEAs, but also because its bifunctional nature allows a clear demonstration of the lipase complete regioselectivity. Despite containing both amino and hydroxyl groups, the enzyme selectively catalyzed aminolysis at the amine functionality, leaving the hydroxyl group untouched. Traditional solvents such as *n*-hexane and toluene—commonly used in similar reactions—are now recognized as hazardous and environmentally harmful.^33^ Consequently, replacing them with greener alternatives is a key step toward improving the sustainability of the process. While selecting an appropriate solvent, it is crucial to consider factors such as polarity, water content, substrate solubility, boiling point, and chemical inertness, as these can significantly influence enzymatic reactions.^47^ To be considered green, a solvent should undergo a comprehensive assessment based on environmental, health, and safety (EHS) metrics, energy consumption during production, and ideally, should be biobased. In this context, *R*-limonene has previously shown promise for the synthesis of OEA *via* CaLB-catalyzed reaction.^14^ However, its high boiling point complicates solvent removal and product recovery. To address this, we adopted an alternative strategy using low-boiling, green solvents that not only align with sustainability criteria but also facilitate product isolation under mild conditions through simple evaporation under reduced pressure. Specifically, 2-methyltetrahydrofuran (2-MeTHF), 2,2,5,5-tetramethyloxolane (TMO), anisole (ANI), phenetole (PHE), and eucalyptol (EUC) were selected not only for their proven compatibility with enzyme-catalyzed transformations, but also due to their potential derivation from biomass.^28,48^ Compared to traditional petrochemical solvents, these alternatives pose fewer health and safety risks.^49^ Notably, 2-MeTHF is predominantly produced from hexoses such as glucose *via* processing of lignocellulosic biomass, and its environmental performance has been validated through life cycle assessment (LCA) studies.^34,50^ TMO, on the other hand, can be readily synthesized through the dehydration of 2,5-dimethylhexane-2,5-diol (DMHDL) using H-beta zeolites at 110 °C for 2 h.^33,36,51^ Although ANI and PHE are naturally present in botanical sources such as aniseed (*Pimpinella anisum*), Chinese woundwort (*Stachys riederi var. japonica*), peach (*Prunus persica*), and *Scutellaria barbata*, they are primarily produced synthetically from phenol—potentially derived from lignin—*via* Williamson ether synthesis.^52,53^ Lastly, EUC, a fragrant terpene ether, can be obtained through fractional distillation of eucalyptol-rich essential oils, offering a renewable and biodegradable solvent option.^54^

Although all tested solvents enabled the efficient synthesis of FAEAs with high isolated yields (TMO: 90%, 2-MeTHF: 93%, PHE: 94%, ANI: 90%, EUC: 95%), EUC was ultimately selected for process implementation based on a combination of performance and practical considerations. EUC is an aprotic, hydrophobic solvent, and prior studies have shown that CaLB exhibits enhanced synthetic activity in less polar media.^7,55,56^ In addition to its favorable physicochemical properties, EUC is readily available in bulk quantities and is more cost-effective than some of the other solvents evaluated in this study, further supporting its suitability for scale-up and sustainable process integration.^57^

### Two-Step Transesterification-Aminolysis Approach: A Sustainable, Intensified Process through SpinChem Technology

Given our group extensive expertise in intensified continuous biocatalytic processes, our initial strategy was to scale up the process in a flow system.^48,58–61^ However, the exceptionally high viscosity of the yeast-derived TAG mixture during its conversion to FAEEs posed major challenges for continuous operations, hampering mass transfer and reactor operability.

To overcome this limitation, we transitioned to SpinChem rotating bed reactor (RBR) technology, which allowed efficient processing of viscous media while maintaining excellent enzyme–substrate contact and preserving catalyst integrity. This shift proved pivotal for both enhancing reaction performance and ensuring the long-term reusability of the immobilized biocatalyst.^62–64^

To maximize catalyst efficiency and reduce material loss, the entire two-step procedure was successfully transferred to the SpinChem RBR (Figure 2). In the first step, TAGs (0.1 M) were transesterified in ethanol using 3 g of Novozym 435 and 3 g of molecular sieves (1:1 w/w) at 55 °C. The reaction reached complete conversion in only 4 h, compared to 24 h of the batch process, leading to a nearly 6-fold improvement in space-time yield (STY batch: 1.3 g L^–1^ h^–1^ vs SpinChem: 7.5 g L^–1^ h^–1^).

**Figure 2:**
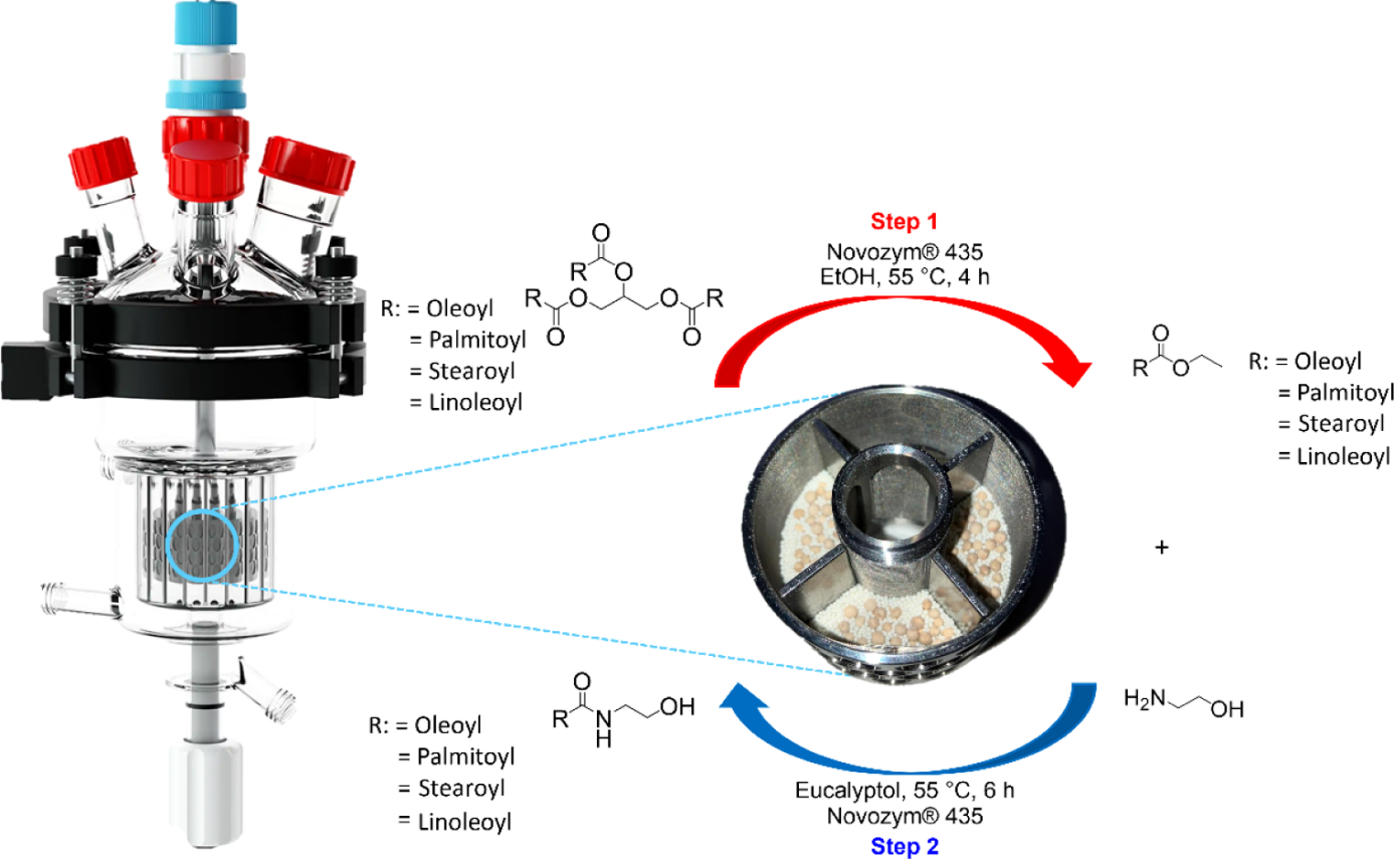
Schematic representation of the intensified process in SpinChem reactor for the lipase-catalyzed synthesis of fatty amides, starting from a TAGs-rich extract and ethanolamine.

After draining the reaction mixture *via* the reactor bottom valve and drying the immobilized enzyme by simple spinning, the crude FAEE mixture—without any intermediate purification—was used directly for the second step: the aminolysis with ethanolamine (1.2 equiv) in EUC. This green solvent was selected for its low toxicity, biobased origin, and ease of removal under reduced pressure. The previously used Novozym 435 and sieves were reemployed for the aminolysis, reaching full conversion within 6 h at 55 °C—drastically reducing the reaction time compared to the conventional batch process, while increasing productivity (STY batch: 1.3/g L^–1^ h^–1^ vs SpinChem: 5.3/g L^–1^ h^–1^).

The obtained FAEAs were carefully analyzed *via* GC-MS, revealing a consistent product distribution: 54% OLA + LEA, 32% PEA, 14% SEA. Due to the coelution of OLA and LEA under the applied GC conditions, complementary LC-MS analysis was performed. The mild ionization settings allowed the detection of the monomolecular ion of each compound in the FAEA mixture, confirming the presence of LEA (see Supporting Information). Importantly, this amide profile was reproducibly obtained even when starting from different TAG batches derived from yeast fermentation, underlining the robustness and reliability of the process. This compositional consistency is critical for applications, particularly when targeting bioactive compounds for nutraceutical or pharmaceutical use and further validates the suitability of this intensified approach for industrial implementation. The SpinChem process achieved complete conversion at lower enzyme-to-substrate ratios (mg_enzyme_/mmol_substrate_) (200 for both transesterification and aminolysis) compared to batch mode (400 and 500, respectively), further highlighting its efficiency. Building on this robustness, the intensified setup also delivered a substantial boost in overall productivity, along with a marked reduction in solvent consumption and energy input. The use of benign solvents like ethanol and EUC—both renewable and environmentally friendly—further enhanced the sustainability profile of the entire process. The corresponding E-factors, accounting for all materials including solvents and reagents, are 30 and 34 for the transesterification and aminolysis steps on a small scale, decreasing to 28 and 30, respectively, on a larger scale, reflecting the slightly improved material efficiency. Moreover, the gentle yet efficient agitation of the rotating bed reactor minimized mechanical stress on the immobilized enzyme compared to conventional stirred batch systems, enabling its reuse over five complete cascade cycles-five transesterification and five aminolysis reactions—with less than 10% loss in enzymatic performance (see Supporting Information).

## Conclusions

This work presents a compelling example of process intensification embedded within a circular bioeconomy framework, demonstrating how integrated biotechnological strategies can convert agri-industrial residues into high-value bioactive compounds. Starting from whey permeate—a major byproduct of dairy processing—used as a cost-effective growth medium, we successfully cultivated *C. oleaginosus* to accumulate intracellular TAGs (48.5% w/w). These microbial lipids, derived *via* fermentation (64 h) enhanced by WCO supplementation (2% w/w), served as renewable feedstocks for the 2-step biocatalytic synthesis of defined FAEAs, namely PEA, OLA, SEA and LEA. Ethanolamine was selected for the biological relevance of the resulting FAEAs as well as to demonstrate the enzyme complete regioselectivity: aminolysis occurred exclusively at the amine, leaving the hydroxyl intact, highlighting both the product value and the robustness of the biocatalytic system. The entire cascade (transesterification followed by aminolysis) was implemented using benign, biobased solvents (ethanol and EUC) and intensified in a SpinChem rotating bed reactor system, which enabled up to a 5-fold reduction in reaction time (from 48 to 10 h), a 2-fold decrease in enzyme-to-substrate ratios (200 vs 400/500), excellent isolated yields (95%), and a 5–7-fold increase in STY (transesterification: batch vs SpinChem: 1.3 g L^–1^ h^–1^ vs 7.5 g L^–1^ h^–1^; aminolysis: batch vs SpinChem RBR: 1.3 g L^–1^ h^–1^ vs 5.3 g L^–1^ h^–1^) compared to conventional batch processes. Moreover, the gentle agitation of the SpinChem reactor minimizing mechanical stress on the enzyme, enabling its reuse over five cascade cycles (each comprising a transesterification followed by an aminolysis) with <10% loss in catalytic performance.

Critically, this intensified approach minimized energy and solvent consumption, allowed complete biocatalyst reuse without detectable activity loss, and ensured a highly reproducible FAEA profile across different yeast lipid batches—meeting key criteria for industrial translation. By transforming low-value residues into bioactive molecules with nutraceutical and therapeutic potential, our platform not only enhances productivity and environmental performance but also exemplifies the principles of circular economy and sustainable manufacturing. This study paves the way for the development of synergistic, biobased formulations of FAEAs from waste-derived resources with broad applications in the fine chemicals, cosmetics, nutraceutical and pharmaceutical sectors.

## Supplementary Material



## Notes

(i) Fermentation process data; (ii) TMO synthesis and characterization; (iii) FAEE and FAEA standard synthesis; (iv) FAEE analysis and characterization (GC-FID and NMR spectra); (v) FAEA analysis and characterization (GC-MS, LC-MS, NMR spectra); (vi) Data on biocatalysts reusability under SpinChem conditions (PDF)

## Abbreviations

(FFAs): Free-fatty acids
(FAEEs): fatty acid ethyl esters
(TAGs): triacylglycerols
(FAEAs): fatty acid ethanolamides
(PEA): palmitoylethanolamide
(OEA): oleoylethanolamide
(SEA): stearoylethanolamide
(LEA): linoleoylethanolamide
(RBR): rotating bed reactor
(2-MeTHF): 2-methyltetrahydrofuran
(TMO): 2,2,5,5-tetramethyloxolane
(ANI): anisole
(PHE): phenetole
(EUC): eucalyptol
(WCO): waste cooking oil
(CW): cheese whey
(WP): whey permeate
