# Supplementary material for: Spinning Green: Lipase-Catalyzed Synthesis of Bioactive Fatty Acid Amides from Renewable Lipid Feedstocks in a Rotating Bed Reactor
Source: ACS Sustain Chem Eng. 2025 Oct 13;13(42):18214–22. doi: 10.1021/acssuschemeng.5c07654 (PMC12570363; doi:10.1021/acssuschemeng.5c07654)
Supplement: Supplementary file 1 [file sc5c07654_si_001.pdf]

Supporting Information

Spinning Green: Lipase-Catalyzed Synthesis of Bioactive Fatty Acid Amides from Renewable Lipid Feedstocks in a Rotating Bed Reactor

Martina Bigliardi,[a] Silvia Donzella,[a] Diana-Ionela Dăescu,[b, c] Alessandro Pellis,[c] Lucia Tamborini,[d] Andrea Pinto,[a] Martina L. Contente[a]*

[a] University of Milan, Department of Food, Nutrition and Environmental Sciences, via Celoria 2, 20133, Milan, Italy.
[b] Politehnica University Timisoara, Faculty of Chemical Engineering, Biotechnologies and Environmental Protection, Vasile Pârvan 6, 300223 Timișoara, Romania
[c] University of Genoa, Department of Chemistry and Industrial chemistry, via Dodecaneso 31, 16146, Genova, Italy.
[d] University of Milan, Department of Pharmaceutical Sciences, via Mangiagalli 25, 20133, Milan, Italy

*corresponding author: martina.contente@unimi.it

Contents

1. ***Fermentation process for lipid production*** 2
2. ***2,2,5,5-Tetramethyloxolane (TMO) preparation*** 2
3. ***Direct enzymatic amidation of carboxylic acids and ethanolamine*** 4
4. ***Ethyl fatty ester and fatty amide standard synthesis*** 4
5. ***Ethyl ester NMR spectra*** 5
6. ***Ethyl ester GC-FID analysis*** 9
7. ***Fatty amides NMR analysis*** 11
8. ***Fatty amide GC-MS analysis*** 15
9. ***Fatty amide LC-MS analysis*** 17
10. ***Catalyst reusability under SpinChem® conditions*** 17
11. ***References*** 18

1. Fermentation process for lipid production

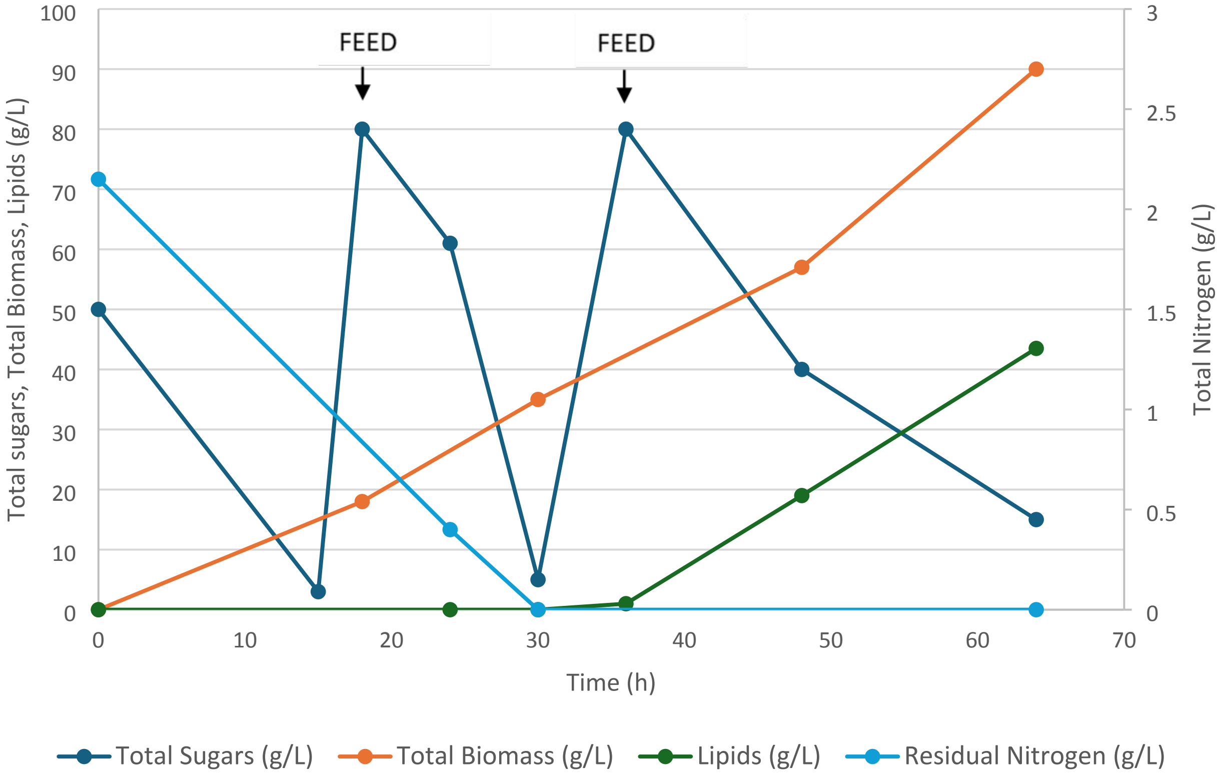

Figure S1: Main fermentation data of *Cutaneotrichosporon oleaginosus* ATCC 20509 process in whey permeate supplemented with urea and WCO. Orange line = total biomass dry weight (DW, g/L); green line = lipid concentration (g/L); light blue line = residual nitrogen (g/L); blue line = residual total sugars (g/L), including lactose (from whey permeate), glucose and fructose from mango syrup feed.

2. 2,2,5,5-Tetramethyloxolane (TMO) preparation

TMO has been synthesized following the procedure reported by Byrne et al.[1] Briefly, 500 g of 2,5-dimethyl-2,5-hexanediol (97%, Merck Life Science srl, Milan, Italy) were melted in a 1L round bottom flask. 1g of hydrogen zeolite beta (Thermo Fisher Scientific S.p.A, Segrate, Milan, Italy) was then added to the molten diol. A Dean-Stark apparatus was set up, and the reaction mixture was stirred at 400 rpm and heated at 130 °C until the reactive distillation was complete. The organic layer was collected, dried over magnesium sulfate, and distilled one more time. The resulting material was stored on molecular sieves until further use.

TMO: ^{1}H NMR (400 MHz, $CDCl_3$) δ (ppm): 1.81 (s, 4H), 1.22 (s, 12H).

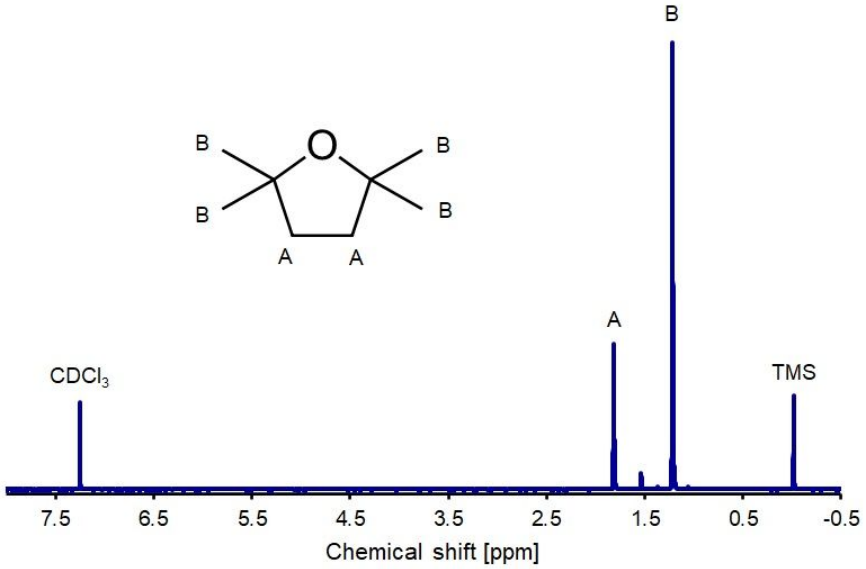

Figure S2: ^{1}H-NMR of TMO.

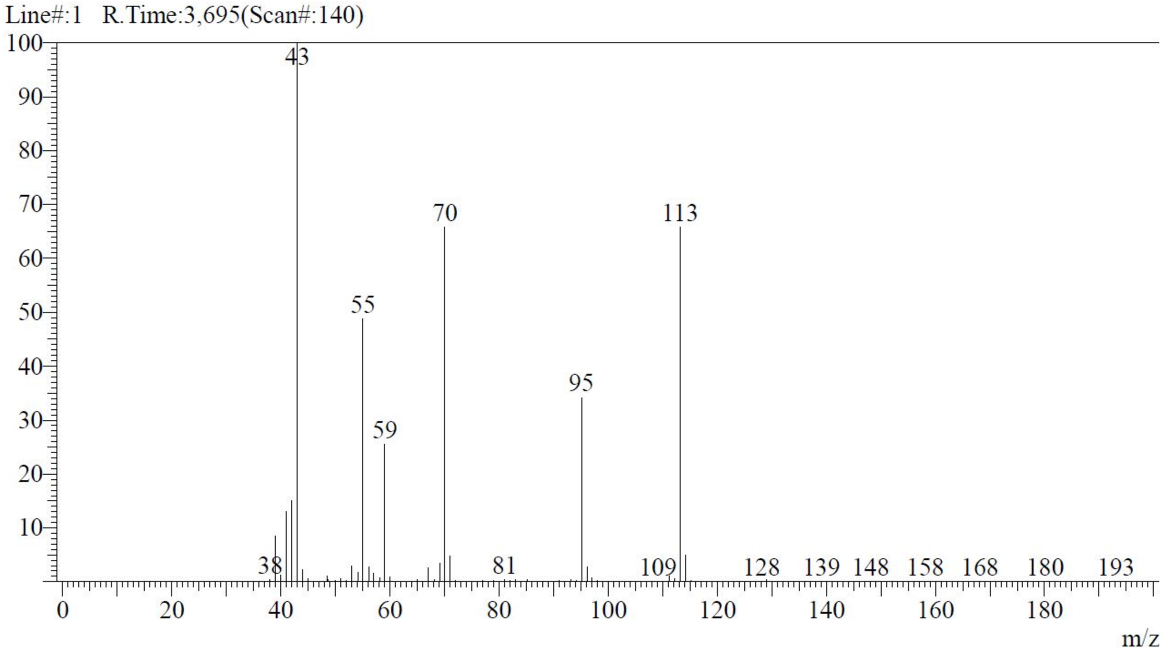

Figure S3: GC-MS analysis of the synthesized TMO.

3. **Direct enzymatic amidation of carboxylic acids and ethanolamine**

Temperature (°C)	Catalyst loading (mg/ml)	Yield (%)		
		n-hexane	Toluene	TMO
55	50	25	28	28
	100	27	30	30
70	50	26	28	30
	100	30	30	30

Table S1: Isolated yield of FAEAs after 24 hours of in different reaction conditions: FFAs (0.1 M, 0.1 mmol) in 1 mL solvent (*n*-hexane or toluene or TMO), ethanolamine (1.2 eq.), Novozym® 435 (50 mg-100 mg) and molecular sieves (1:1 w:w) at 150 rpm, 55-70 °C.

4. **Ethyl fatty ester and fatty amide standard synthesis**

Fatty acid standards: oleic acid, palmitic acid, stearic acid, linoleic acid were purchased from Merck Life Science srl (Milan, Italy).

Ethyl fatty ester standards: ethyl fatty esters were synthetized following the procedure reported by Quintana et al. (2016).[1] Briefly: CaL B (400 mg) was added to a solution of fatty acid (0.1 M, 1 mmol) in ethanol (10 mL). The suspension was shaken at 200 rpm and 55 °C. Once the reaction was finished (24 h), the enzyme was filtered off. After evaporation of the solvent under reduced pressure and column chromatography (silica gel), eluted with a gradient method *n*-hexane/EtOAc (95:5), a powder or an oily residue was obtained with the following yields: ethyl palmitate 85%, ethyl oleate 90%, ethyl stearate 85% and ethyl linoleate 90%.

Fatty amide standards: fatty amides were synthesized by adapting the protocol described by Quintana et al. (2016).[2] Briefly: to a solution of ethyl fatty esters (0.1 M, 1 mmol) in *n*-hexane (10 mL), CaL B (600 mg) and ethanolamine (1.2 eq) were added. The suspension was shaken at 200 rpm and 55 °C. Once the reaction was finished (48 h), the enzyme was filtered off and the solvent evaporated under reduced pressure. The residue was purified by column chromatography (silica gel) employing mixtures of *n*-hexane/EtOAc as eluent (9:1–100%), to obtain a pure compound as a colourless oil or white powder with the following yields: *N*-palmitoylethanolamide 15%, *N*-oleoylethanolamide 65%, *N*-stearylethanolamide 18% and *N*-linoleylethanolamide 70%.

5. Ethyl ester NMR spectra

Ethyl oleate: ^{1}H NMR (400 MHz, $CDCl_3$) δ (ppm): 5.40 – 5.28 (m, 2H), 4.12 (q, *J* = 7.1 Hz, 2H), 2.28 (t, *J* = 7.5 Hz, 2H), 2.07 – 1.94 (m, 4H), 1.75 – 1.45 (m, 2H), 1.30 – 1.23 (m, 23H), 0.88 (t, *J* = 6.8 Hz, 3H). ^{13}C NMR (100 MHz, $CDCl_3$) δ (ppm): 173.9, 130.0, 129.8, 60.1, 34.4, 31.9, 29.8, 29.7, 29.5, 29.3 (x2), 29.2, 29.1, 29.0, 27.2, 27.1, 24.9, 22.7, 14.2, 14.1.

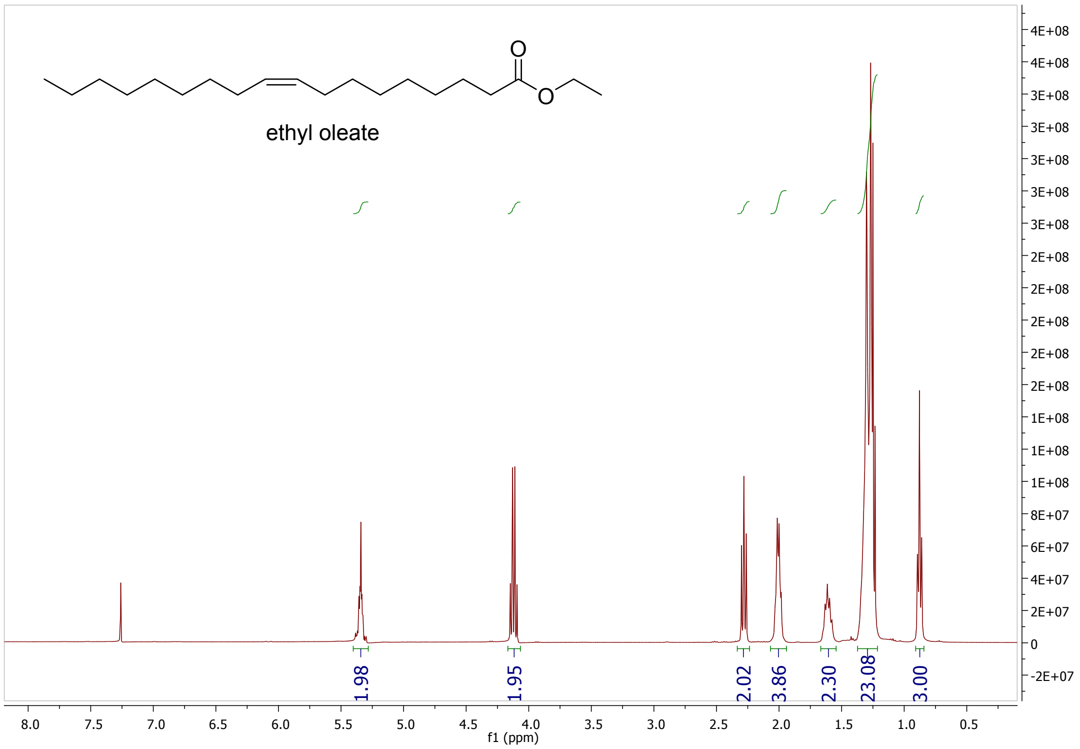

Figure S4: ^{1}H-NMR ethyl oleate.

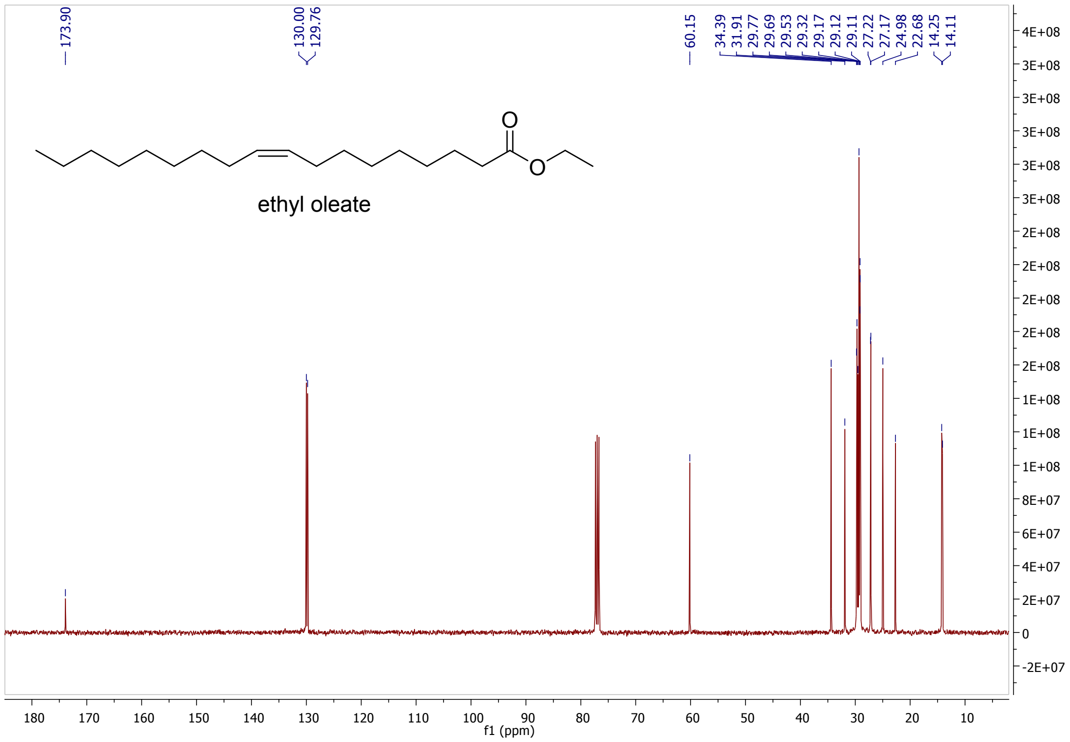

Figure S5: ^{13}C -NMR ethyl oleate.

Ethyl palmitate: ^{1}H NMR (400 MHz, $CDCl_3$) δ (ppm): 4.12 (q, *J* = 7.1 Hz, 2H), 2.27 (t, *J* = 7.5 Hz, 2H), 1.63 – 1.57 (m, 2H), 1.32 – 1.21 (m, 27H), 0.87 (t, *J* = 6.8 Hz, 3H). ^{13}C NMR (100 MHz, $CDCl_3$) δ (ppm): 173.9, 60.1, 34.4, 31.9, 29.7 (x4), 29.6 (x3), 29.6, 29.5, 29.3, 29.3, 29.1, 24.9, 22.7, 14.2, 14.0.

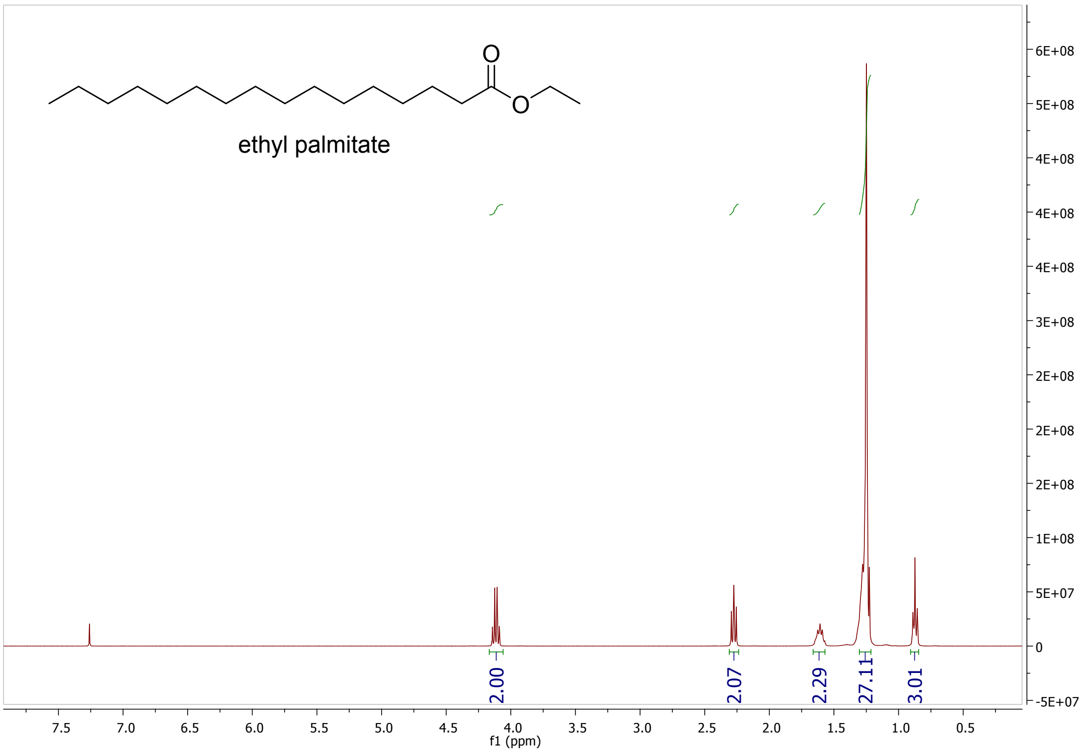

Figure S6: ^{1}H-NMR ethyl palmitate.

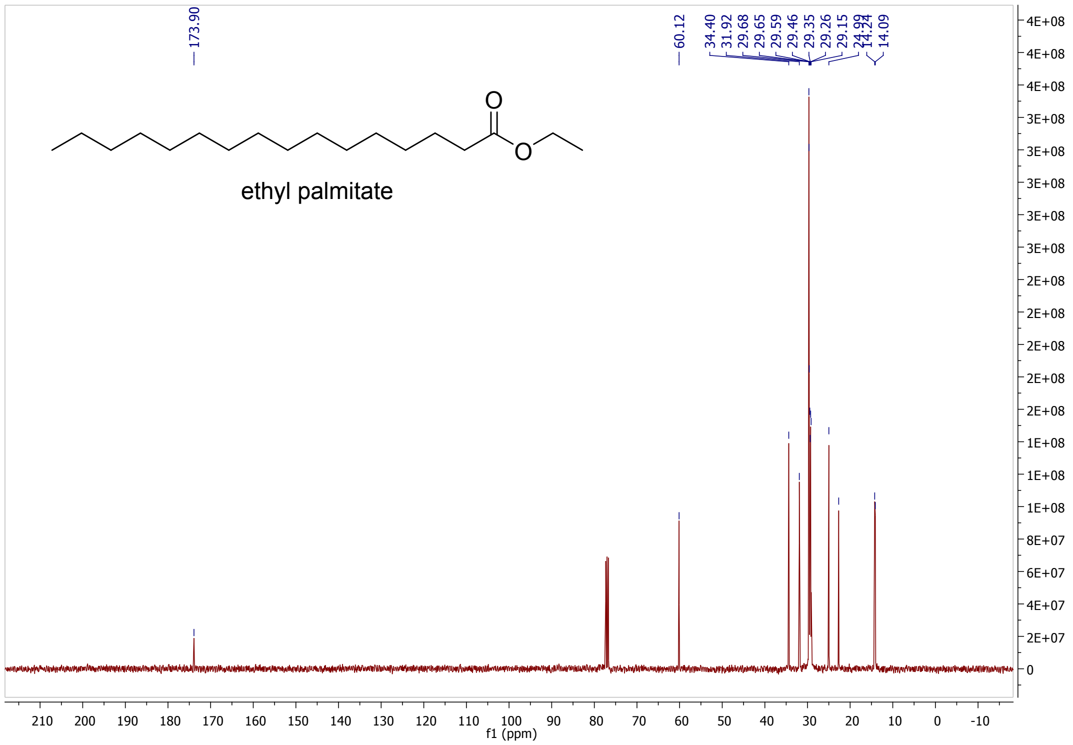

Figure S7: ^{13}C-NMR ethyl palmitate.

Ethyl stearate: ^{1}H NMR (400 MHz, $CDCl_3$) δ (ppm): 4.11 (q, J = 7.1 Hz, 2H), 2.27 (t, *J* = 7.5 Hz, 2H), 1.67 – 1.54 (m, 2H), 1.38 – 1.10 (m, 32H), 0.87 (t, *J* = 6.8 Hz, 3H).^{13}C NMR (100 MHz, $CDCl_3$) δ (ppm): 173.9, 60.1, 34.4, 31.9, 29.7 (x7), 29.6, 29.5, 29.4, 29.3, 29.1, 24.9, 22.7, 14.2, 14.1.

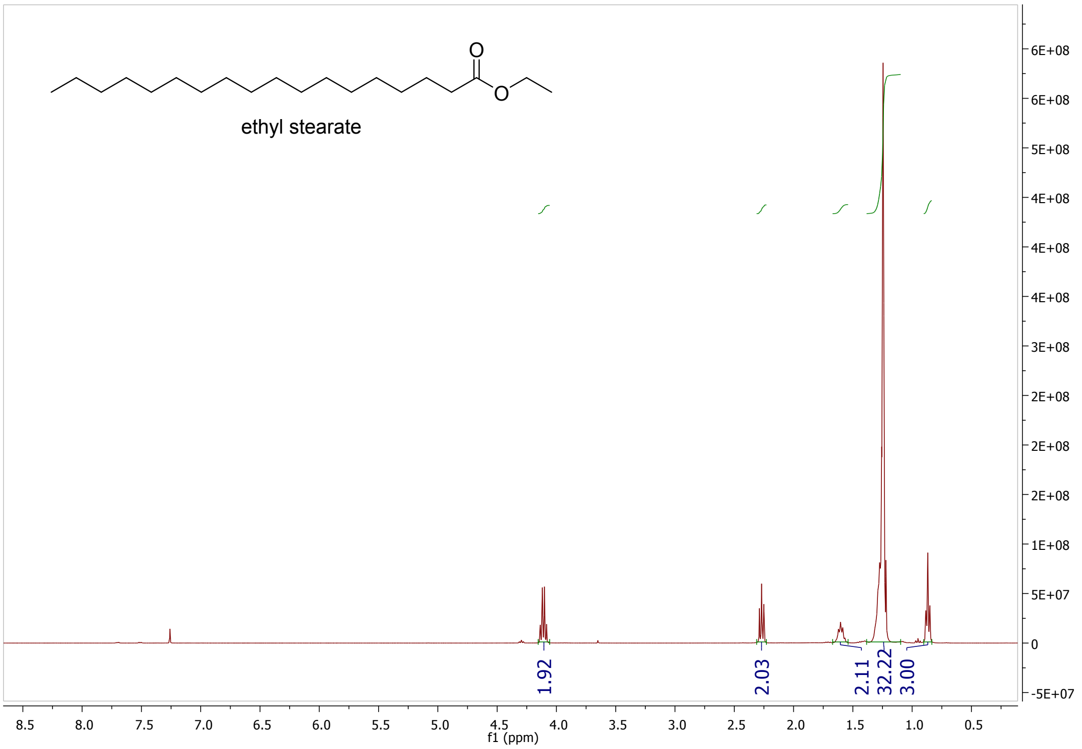

Figure S8: ^{1}H-NMR ethyl stearate.

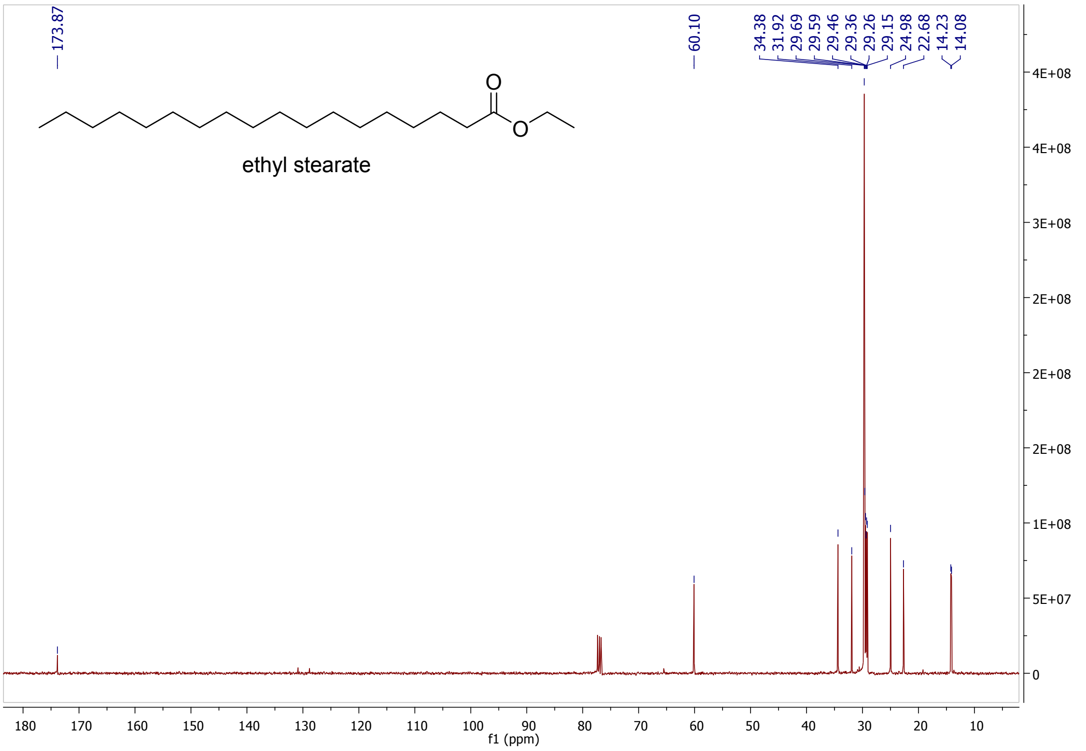

Figure S9: ^{13}C-NMR ethyl stearate.

Ethyl linoleate: ^{1}H NMR (400 MHz, $CDCl_3$) δ (ppm): 5.45 – 5.25 (m, 4H), 4.12 (q, *J* = 7.1 Hz, 2H), 2.78 – 2.72 (m, 2H), 2.28 (t, *J* = 7.5 Hz, 2H), 2.07 – 2.02 (m, 4H), 1.63 – 1.57 (m, 2H), 1.42 – 1.27 (m, 14H), 1.25 (t, *J* = 7.1 Hz, 3H), 0.88 (t, *J* = 6.8 Hz, 3H). ^{13}C NMR (100 MHz, $CDCl_3$) δ (ppm): 173.9, 130.2, 130.0, 128.04, 127.9, 60.1, 34.4, 31.5, 29.6, 29.3, 29.2, 29.1 x2, 27.2 x2, 25.6, 24.9, 22.6, 14.3, 14.1.

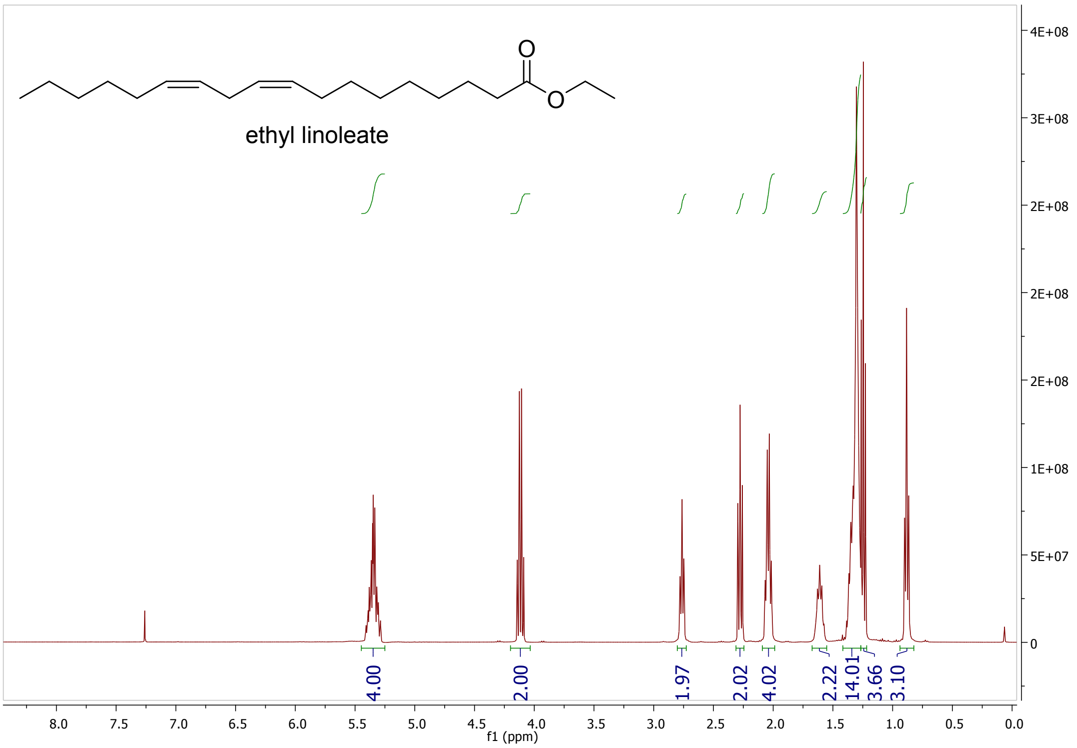

Figure S10: ^{1}H-NMR ethyl linoleate.

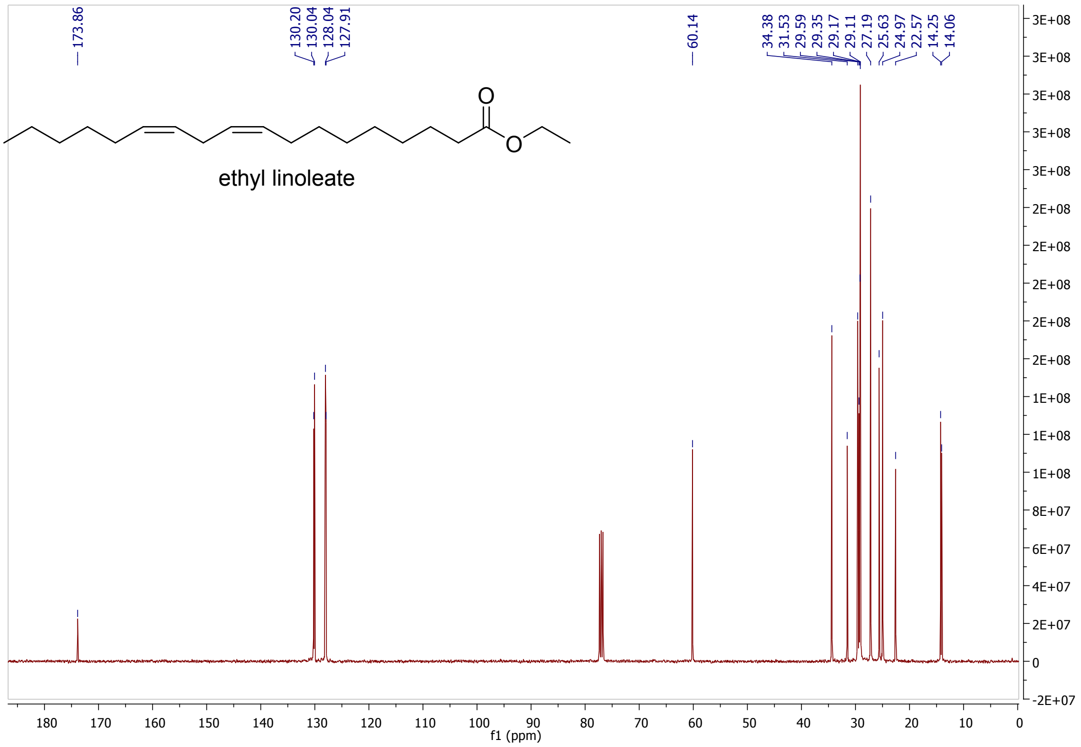

Figure S11: ^{13}C-NMR ethyl linoleate.

6. Ethyl ester GC-FID analysis

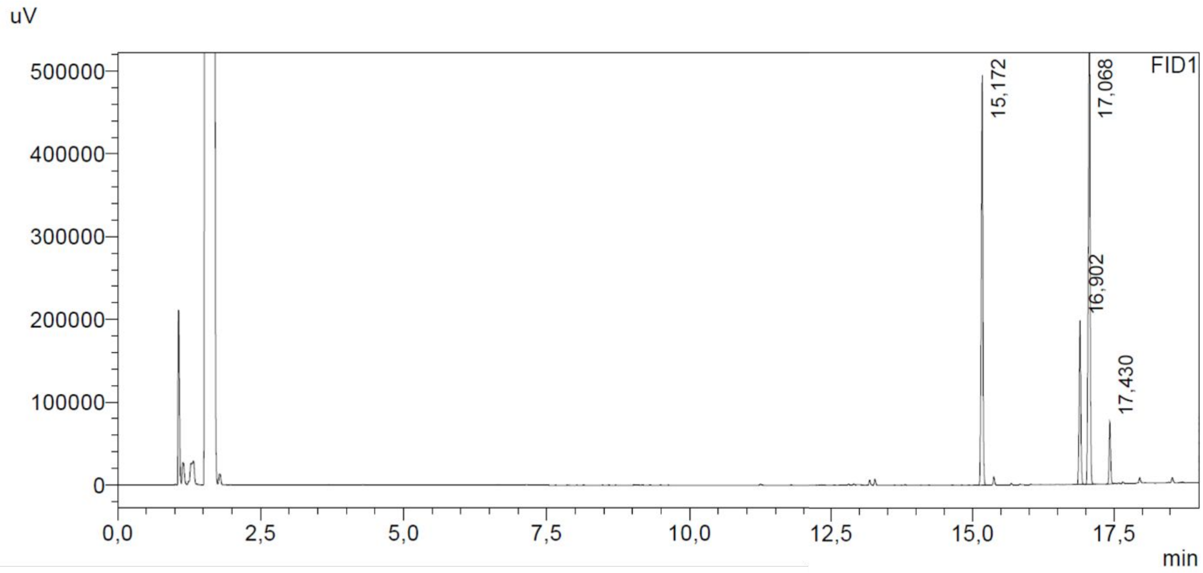

Peak	Name	Ret. Time	Perc. (%)
1	ethyl palmitate	15.19	31.6
2	ethyl stearate	16.92	13.8
3	ethyl oleate	17.09	50.1
4	ethyl linoleate	17.43	4.5

Figure S12: GC-FID chromatogram of transesterification of TAGs into ethyl fatty esters.

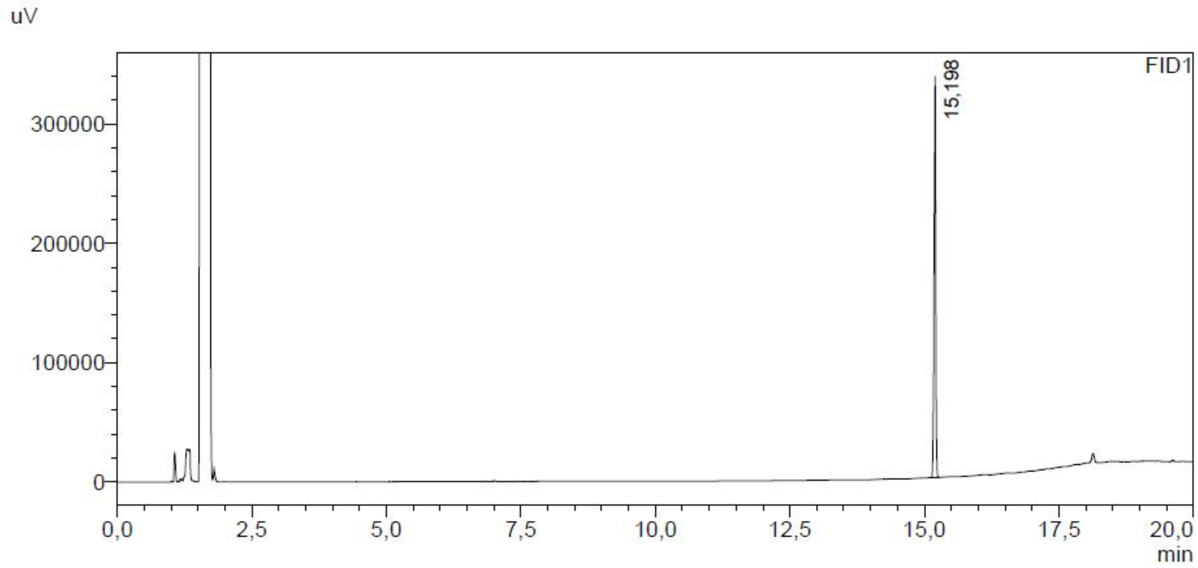

Figure S13: GC-FID chromatogram ethyl palmitate.

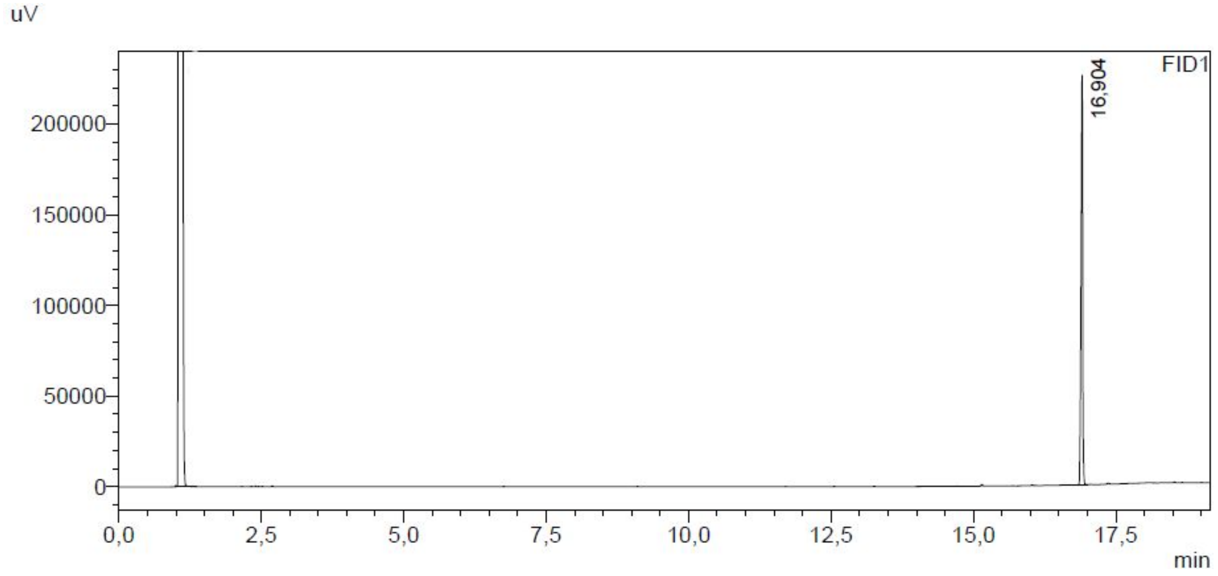

Figure S14: GC-FID chromatogram ethyl stearate.

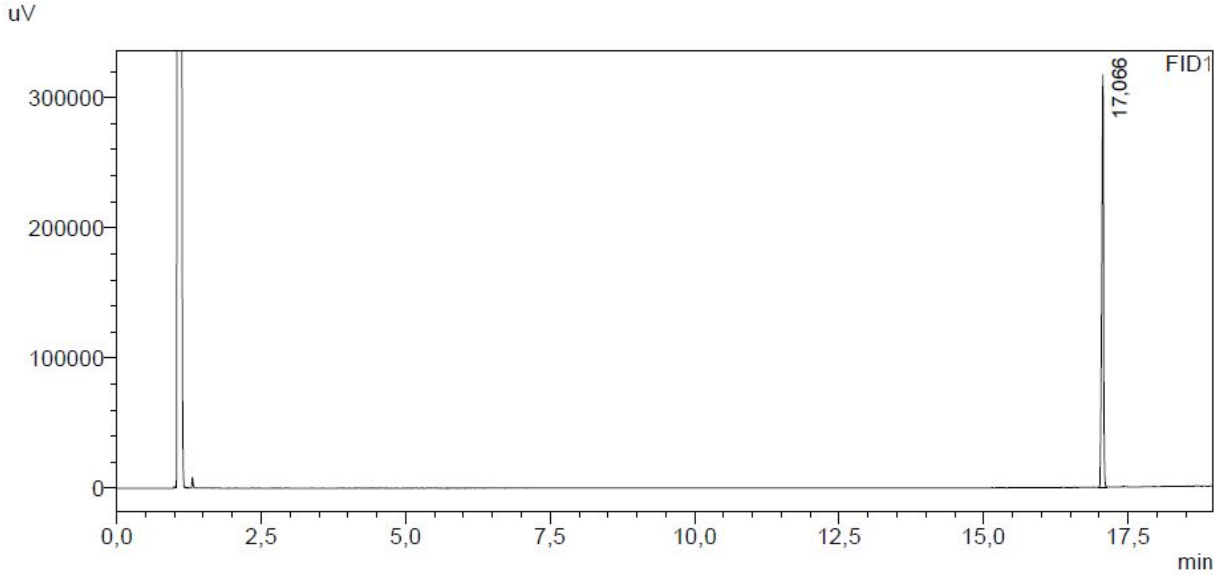

Figure S15: GC-FID chromatogram ethyl oleate.

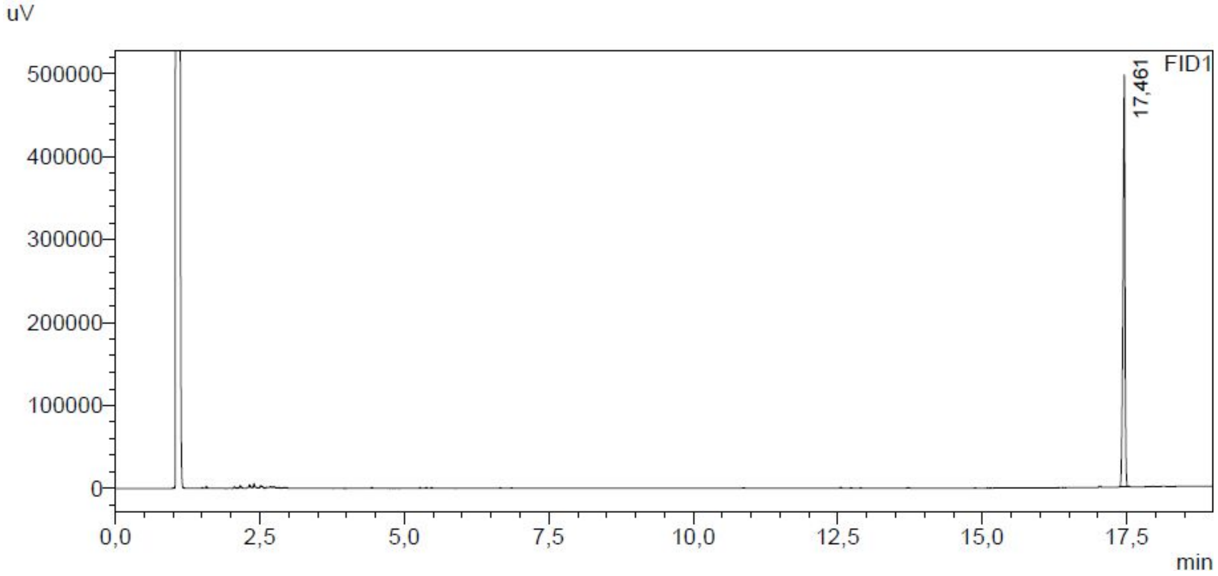

Figure S16: GC-FID chromatogram ethyl linoleate.

7. Fatty amides NMR analysis

***N*-oleoylethanolamide:** ^{1}H NMR (400 MHz, $CDCl_3$) δ (ppm): 6.35 (bs, 1H), 5.43 – 5.22 (m, 2H), 3.74 – 3.63 (m, 2H), 3.56 (bs, 1H), 3.43 – 3.30 (m, 2H), 2.20 (t, *J* = 7.5 Hz, 2H), 2.07 – 1.96 (m, 4H), 1.61 – 1.58 (m, 2H), 1.44 – 1.14 (m, 20 H), 0.86 (t, *J* = 6.8 Hz, 3H). ^{13}C NMR (100 MHz, $CDCl_3$) δ (ppm): 174.6, 130.0, 129.7, 62.0, 42.3, 36.6, 31.9, 29.7, 29.7, 29.5, 29.3, 29.3, 29.2 (x3), 27.2, 27.2, 25.7, 22.7, 14.1 according to Ottria R. et al.[3]

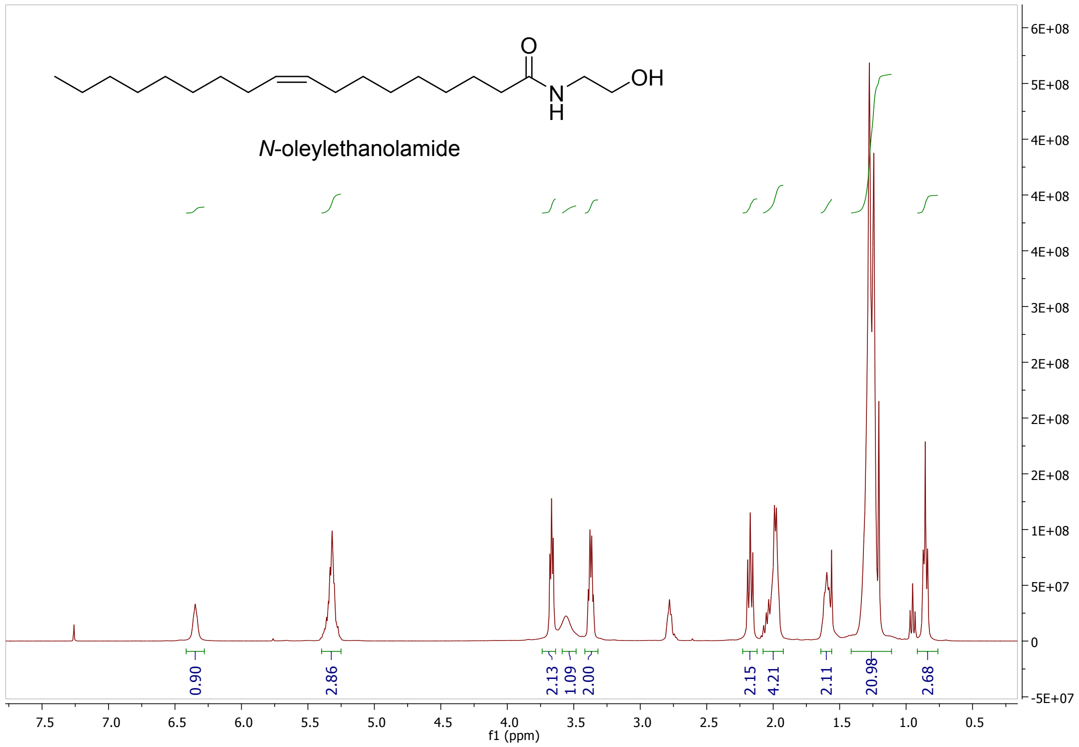

Figure S17: ^{1}H-NMR *N*-oleoylethanolamide.

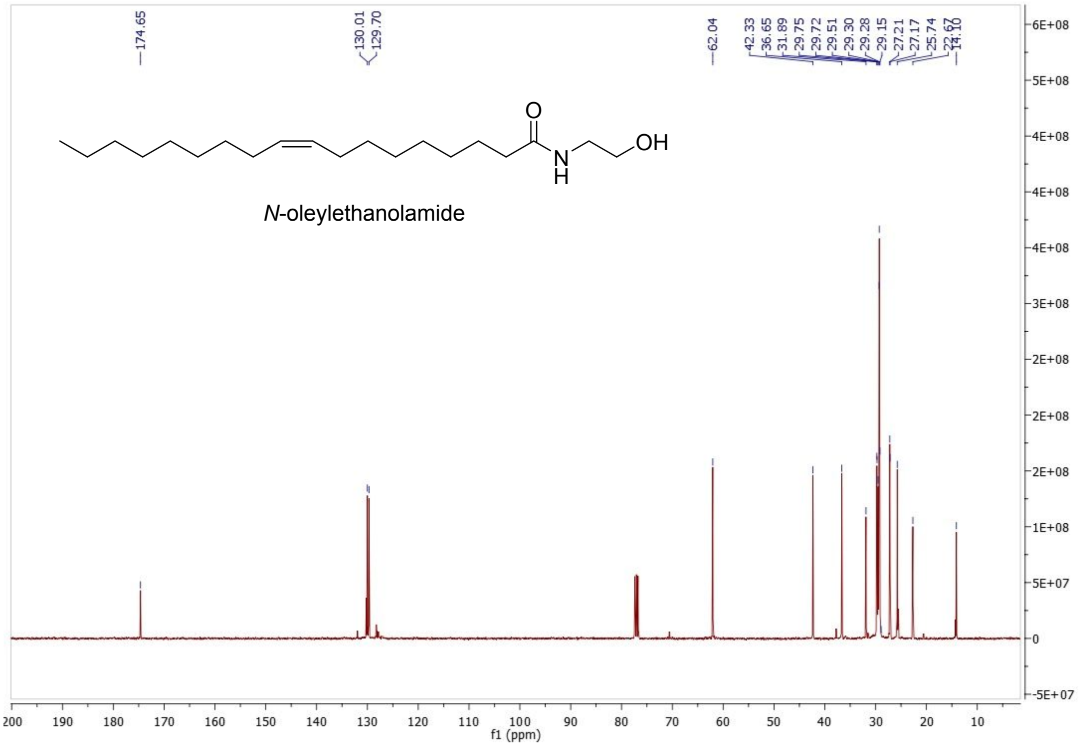

Figure S18: ^{13}C-NMR *N*-oleoylethanolamide.

***N*-palmitoylethanolamide**: ^{1}H NMR (400 MHz, $CDCl_3$) δ (ppm): 5.99 (bs, 1H), 3.73 – 3.71 (m, 2H), 3.48 (s, 1H), 3.44 – 3.40 (m, 2H), 2.20 (t, *J* = 7.5 Hz, 2H), 1.69 – 1.57 (m, 2H), 1.28 – 1.25 (m 24H), 0.87 (t, *J* = 6.8 Hz, 3H). ^{13}C NMR (100 MHz, $CDCl_3$) δ (ppm): 174.6, 62.6, 42.5, 36.7, 31.9, 29.8, 29.7, 29.6, 29.5, 29.4 (x3), 29.3 (x3), 25.7, 22.7, 14.1 according to Ottria R. et al.[3]

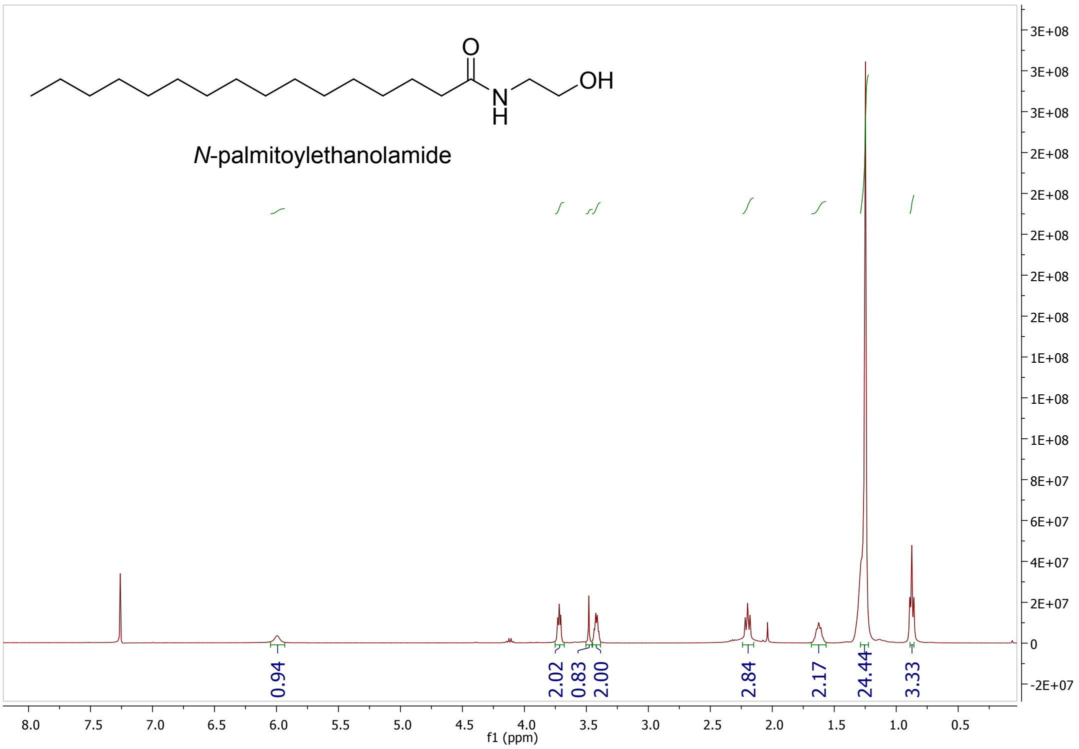

Figure S19: ^{1}H-NMR *N*-palmitoylethanolamide.

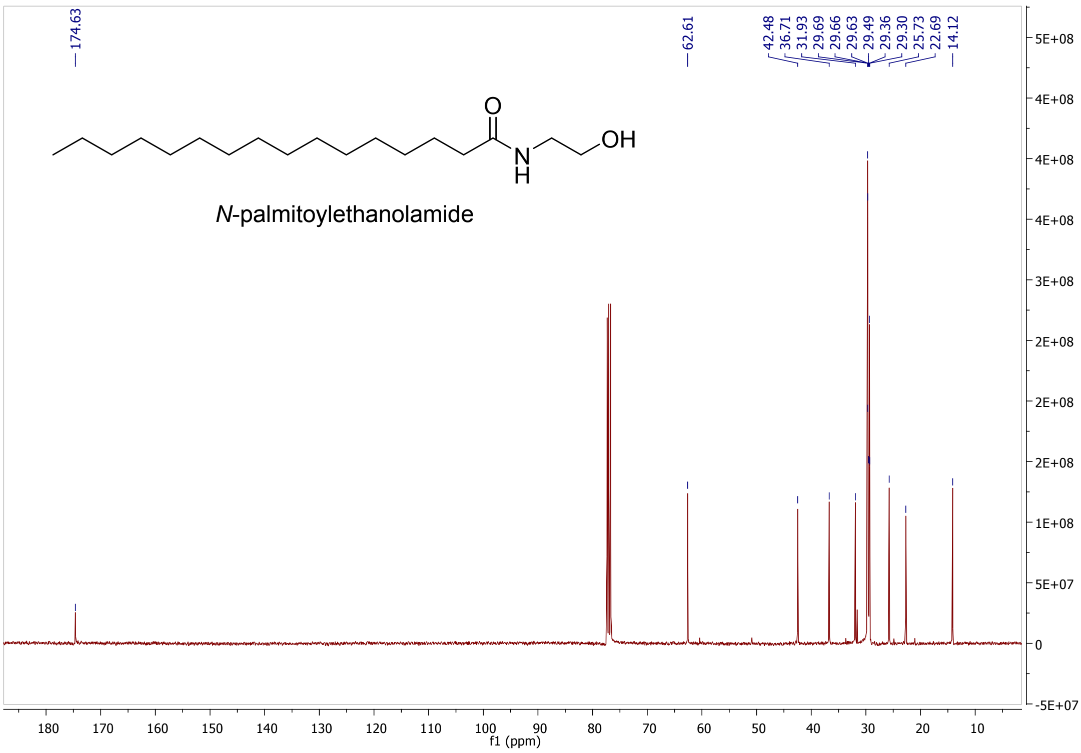

Figure S20: ^{13}C-NMR *N*-palmitoylethanolamide.

***N*-stearylethanolamide:** ^{1}H NMR (400 MHz, $CDCl_3$) δ (ppm): 5.91 (bs, 1H), 3.74 – 3.71 (m, 2H), 3.44 – 3.40 (m, 2H), 2.64 (bs, 1H), 2.20 (t, *J* = 7.5 Hz, 2H), 1.65 – 1.62 (m, 2H), 1.28 – 1.25 (m, 28H), 0.88 (t, *J* = 6.8 Hz, 3H). ^{13}C-NMR (100 MHz, $CDCl_3$) δ (ppm): 174.6, 70.7, 62.3, 42.3, 37.8 (x2), 36.7, 31.9, 29.7, 29.5 (x2), 29.4 (x4), 29.2, 29.1, 25.7, 22.7, 14.1, according to Ottria R. et al.[3]

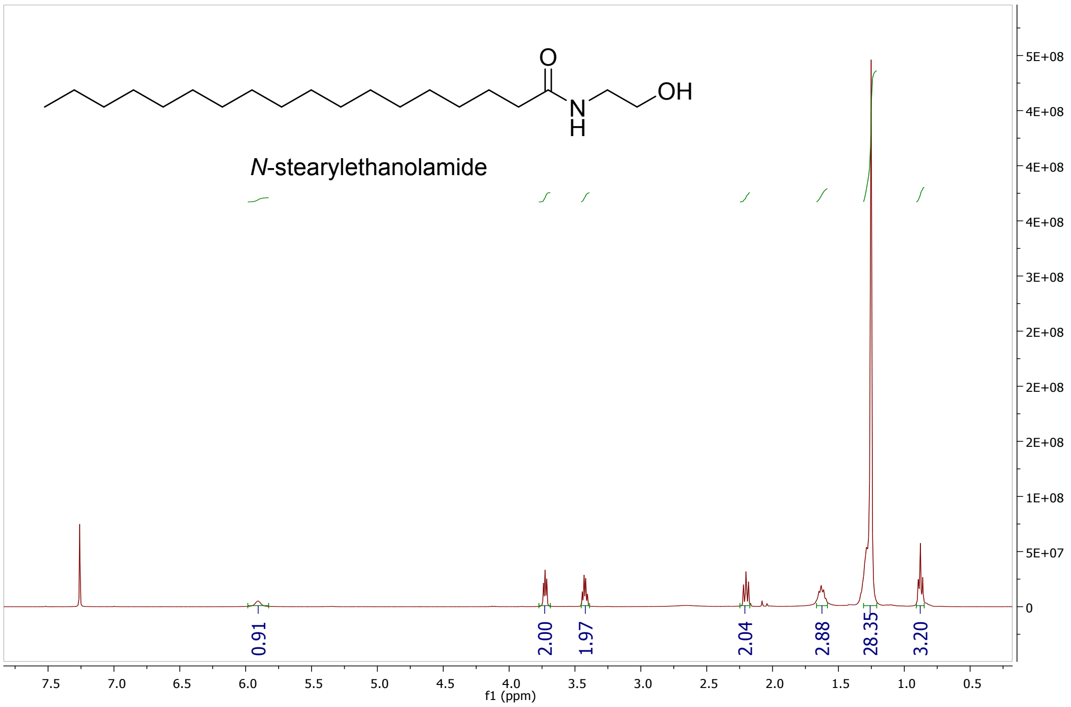

Figure S21: ^{1}H-NMR *N*-stearylethanolamide.

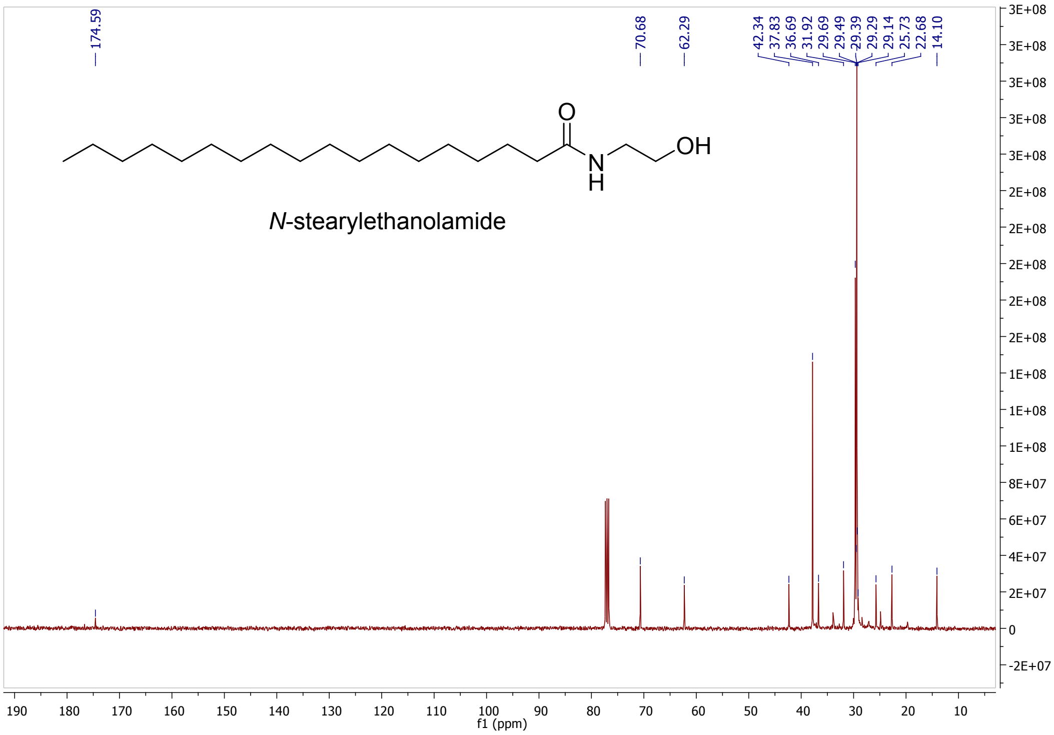

Figure S22: ^{13}C-NMR *N*-stearylethanolamide.

***N*-linolenoylethanolamide:** ^{1}H NMR (400 MHz, $CDCl_3$) δ (ppm): 6.13 (bs, 1H), 5.48 – 5.22 (m, 4H), 3.71 – 3.68 (m, 2H), 3.42 – 3.38 (m, 2H), 2.81 – 2.67 (m, 2H), 2.20 (t, J = 7.5 Hz, 2H), 2.08 – 1.97 (m, 4H), 1.68 – 1.53 (m, 2H), 1.42 – 1.18 (m, 14H), 0.88 (t, J = 6.8 Hz, 3H). ^{13}C NMR (100 MHz, $CDCl_3$) δ (ppm): 174.6, 131.9, 130.3, 128.3, 127.7, 62.5, 42.4, 37.8, 36.7, 29.6, 29.4, 29.3 (x2), 29.1, 28.1, 27.2, 25.7, 25.6, 25.5, 14.3 according to Ottria R. et al.[3]

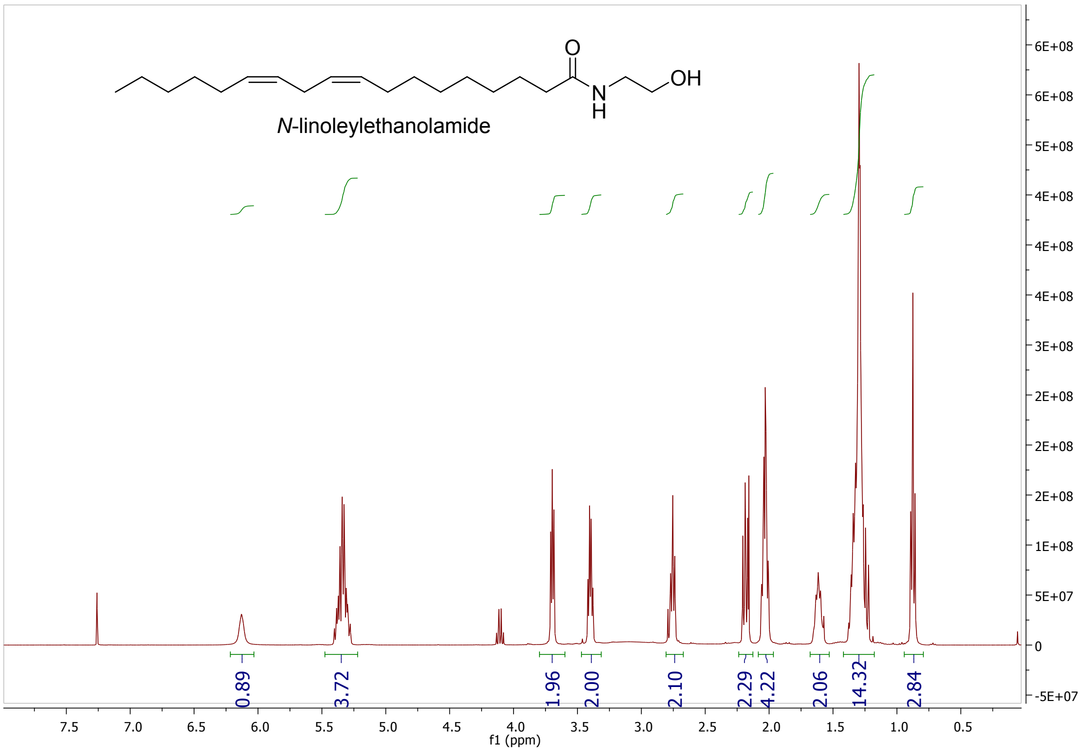

Figure S23: ^{1}H-NMR *N*-linolenoylethanolamide.

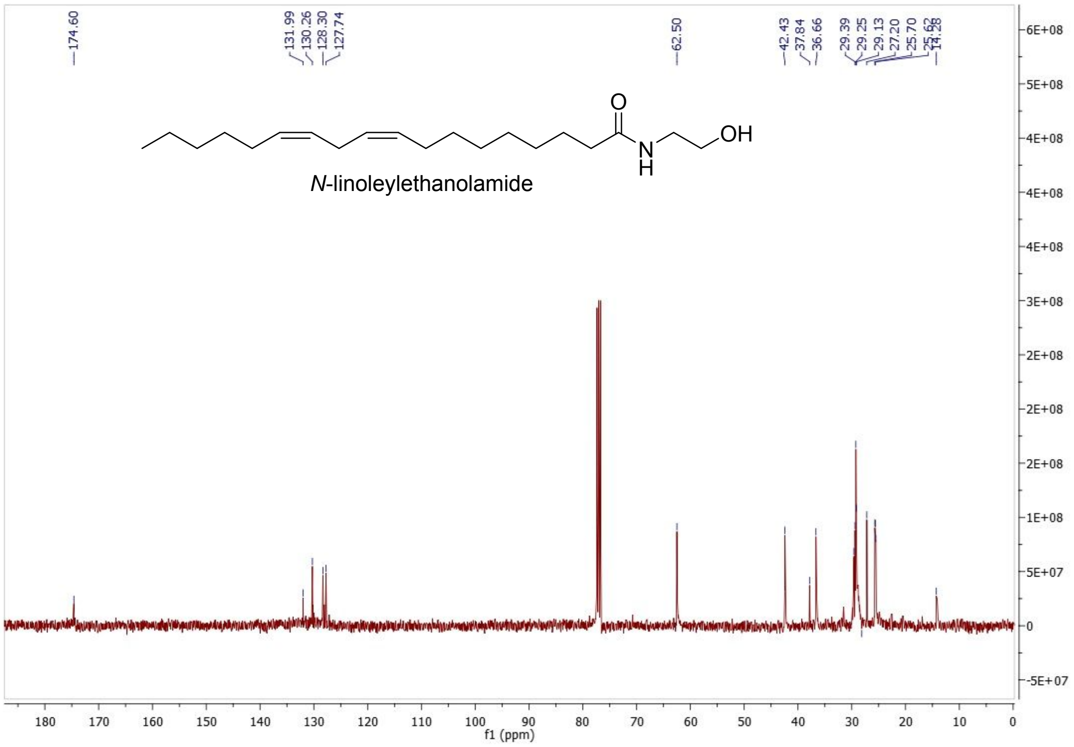

Figure S24: ^{13}C-NMR *N*-linolenoylethanolamide.

8. Fatty amide GC-MS analysis

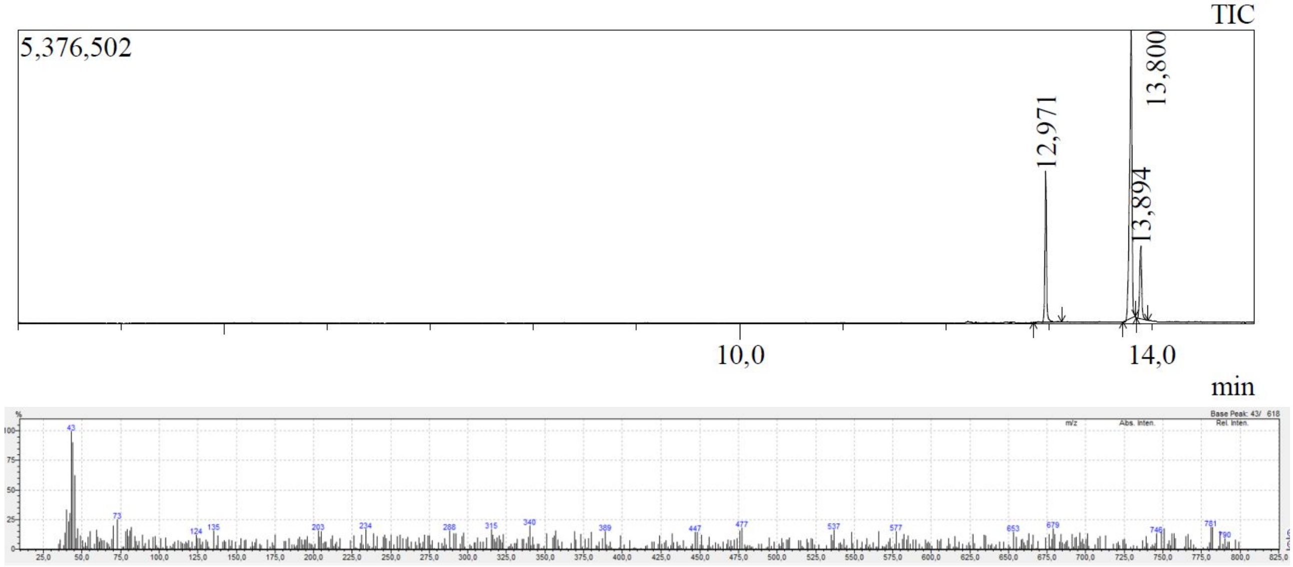

Peak	Name	Ret. Time	Perc. (%)
1	*N*-palmitoylethanolamide	12.97	31.8
2	*N*-oleoylethanolamide + *N*-linolenoylethanolamide	13.80	54.3
3	*N*-stearylethanolamide	13.89	13.9

Figure S25: GC-MS chromatogram and mass spectrum of FAEA mixture

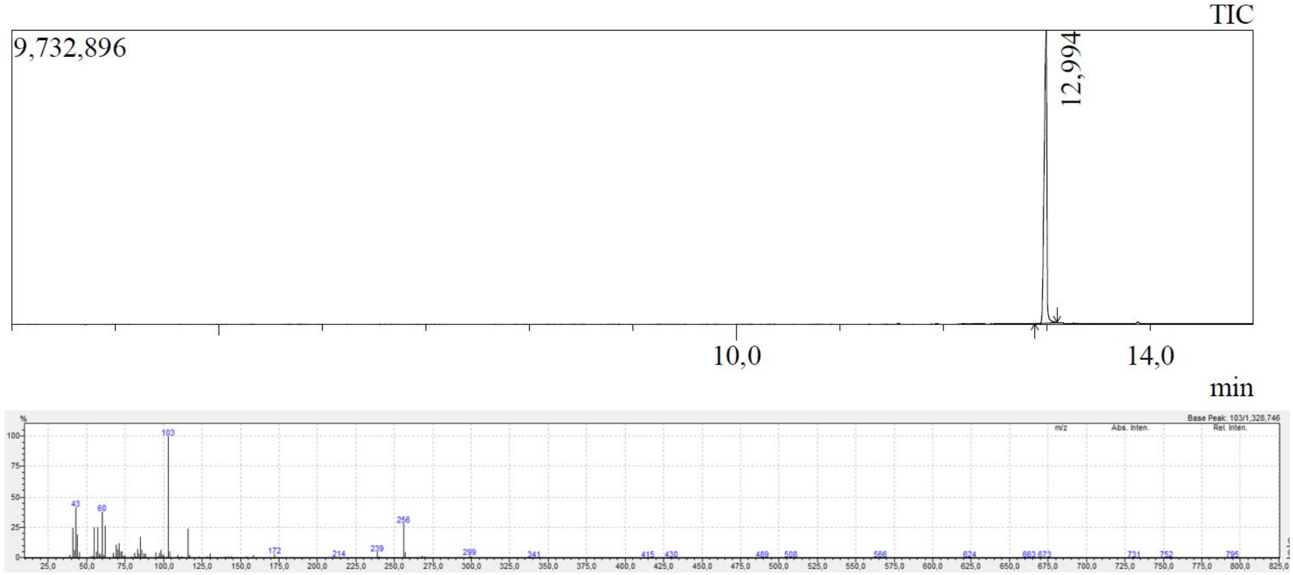

Figure S26: GC-MS chromatogram and mass spectrum of *N*-palmitoylethanolamide

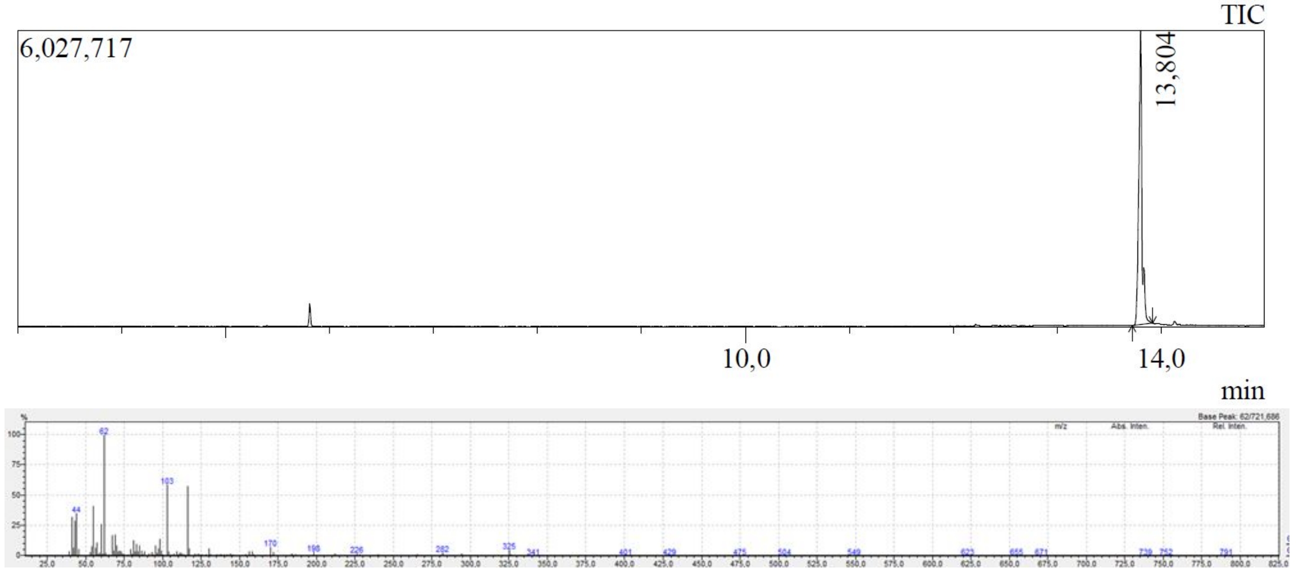

Figure S27: GC-MS chromatogram and mass spectrum of *N*-oleoylethanolamide

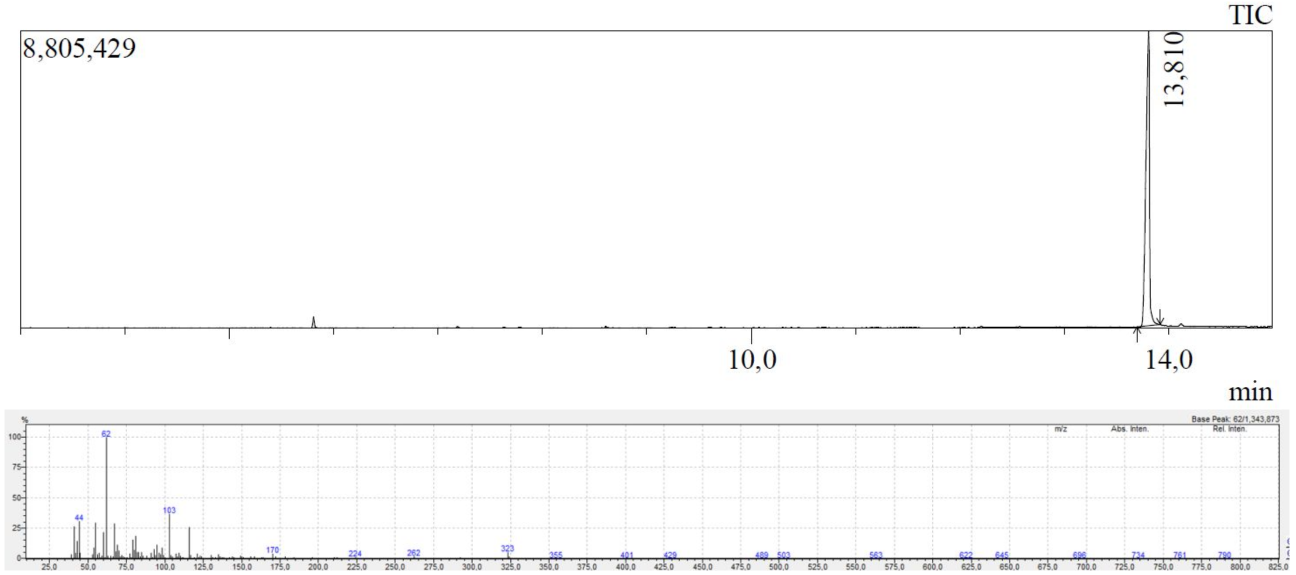

Figure S28: GC-MS chromatogram and mass spectrum of *N*-linolenoylethanolamide

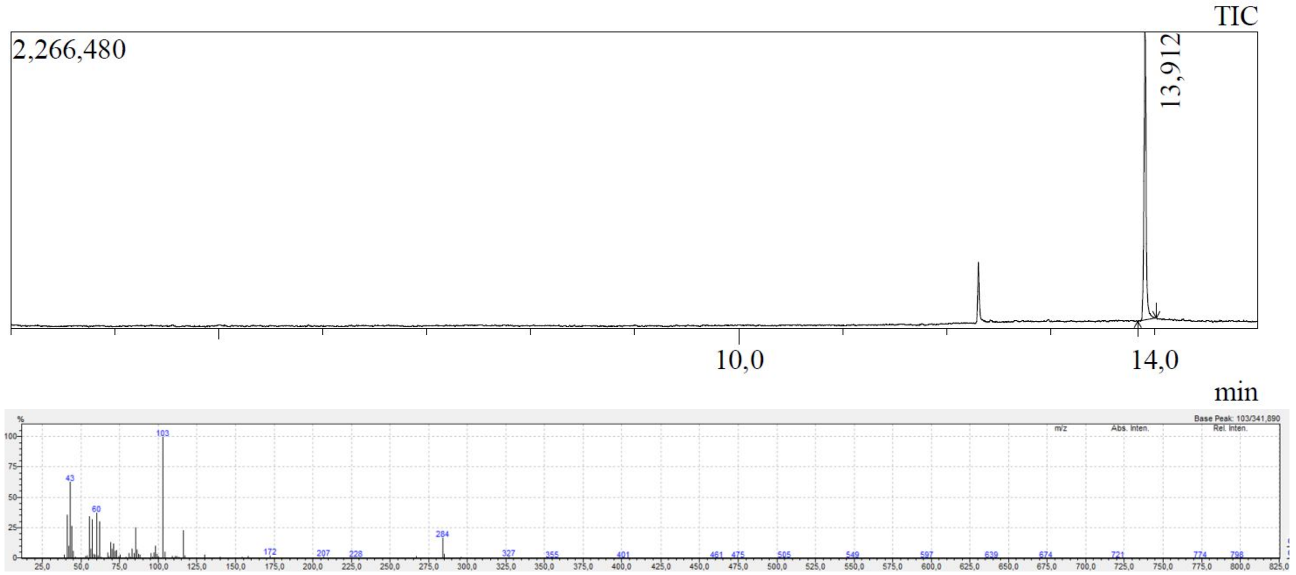

Figure S29: GC-MS chromatogram and mass spectrum of *N*-stearylethanolamide

9. Fatty amide LC-MS analysis

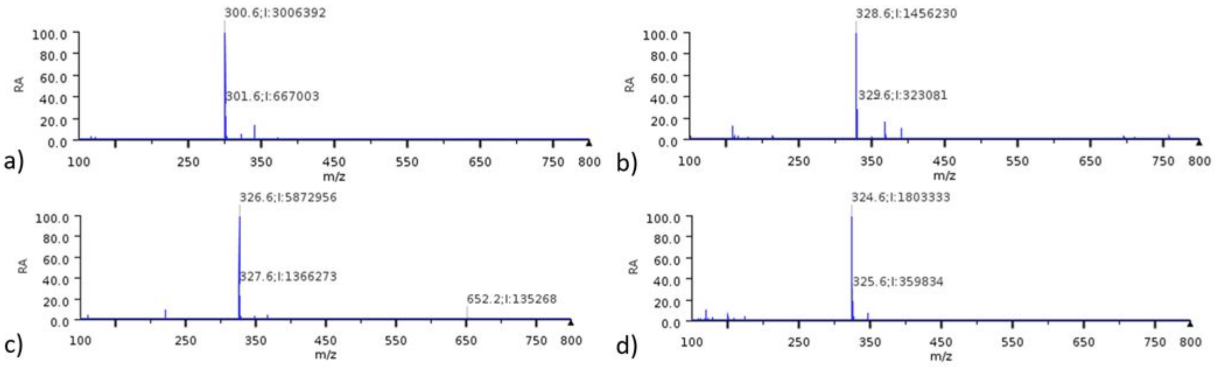

Figure S30: LC-MS spectra of FAEA mixture: a) *N*-palmitoylethanolamide b) *N*-stearylethanolamide c) *N*-oleoylethanolamide d) *N*-linoleoylethanolamide

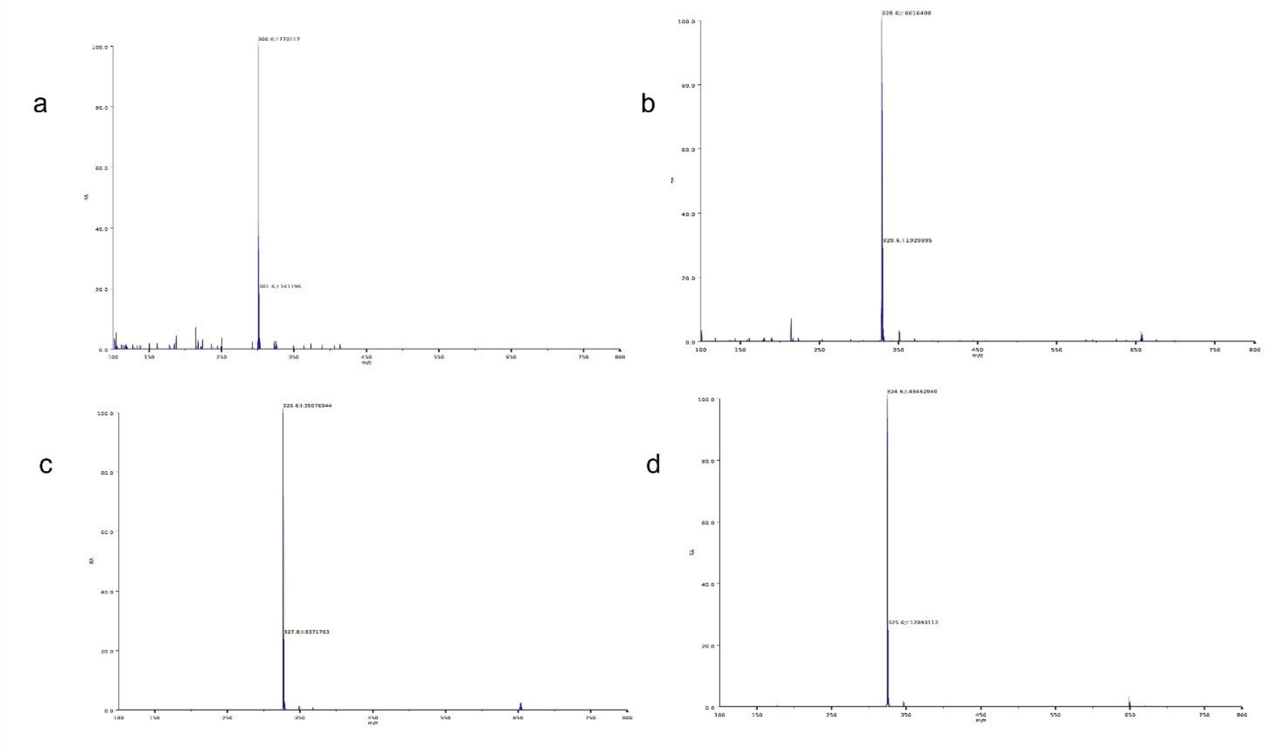

Figure S31: LC-MS spectra: a) *N*-palmitoylethanolamide standard b) *N*-stearylethanolamide standard c) *N*-oleoylethanolamide standard d) *N*-linolenoylethanolamide standard

10. Catalyst reusability under SpinChem® conditions

The catalyst reusability has been tested under SpinChem® technology alloing for five complete cascade cycles—comprising five transesterification and five aminolysis reactions—with less than 10% loss in isolated yield.

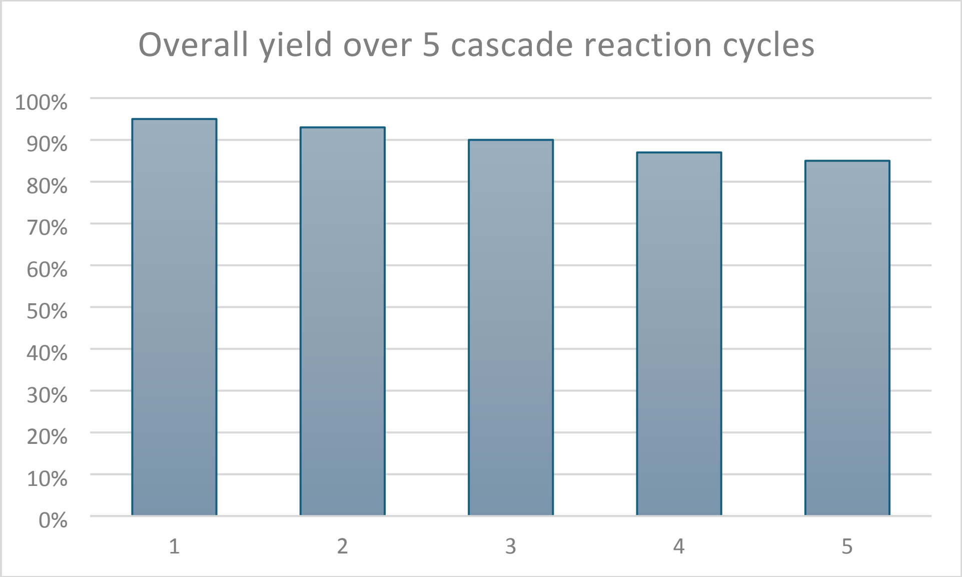

Figure S32: Catalyst reusability over 5 cascade reaction cycles. Cascade reaction cycles = transesterification followed by aminolysis biotransformation. Overall yield = isolated yield calculated over the 2-step reaction.

11. References

1. Byrne F. P.; Clark J. H.; Angelici C.; de Jong E.; Farmer T. J. Greenness Assessment and Synthesis for the Bio-Based Production of the Solvent 2,2,5,5-Tetramethyloxolane (TMO). *Sustain Chem* 2021, 2, 392-406. https://doi.org/10.3390/suschem2030023.

2. Quintana, P. G.; García Liñares, G.; Chanquia, S. N.; Gorojod, R. M.; Kotler, M. L.; Baldessari, A. Improved Enzymatic Procedure for the Synthesis of Anandamide and N-Fatty Acylalkanolamine Analogues: A Combination Strategy to Antitumor Activity. *Eur J Org Chem* 2016, **2016**, 518–528. https://doi.org/10.1002/ejoc.201501263.

3. Ottria, R.; Casati, S.; Ciuffreda, P. 1H, 13C and 15N NMR Assignments for N- and O-Acylethanolamines, Important Family of Naturally Occurring Bioactive Lipid Mediators. *Magnetic Resonance in Chemistry* **2012**, *50* (12), 823–828. https://doi.org/10.1002/MRC.3891.
